# 6’-sialyllactose ameliorates the ototoxic effects of the aminoglycoside antibiotic neomycin in susceptible mice

**DOI:** 10.3389/fimmu.2023.1264060

**Published:** 2023-12-07

**Authors:** Tawfik Abou Assale, Thomas Kuenzel, Tamara Schink, Anahita Shahraz, Harald Neumann, Christine Klaus

**Affiliations:** ^1^ Neural Regeneration, Institute of Reconstructive Neurobiology, Medical Faculty and University Hospital of Bonn, University of Bonn, Bonn, Germany; ^2^ Auditory Neurophysiology, Department of Chemosensation, Institute for Biology II, RWTH Aachen University, Aachen, Germany

**Keywords:** neuroinflammation, sialylation, 6’-sialyllactose, neomycin, hearing loss, macrophages

## Abstract

Sialic acids are terminal sugars of the cellular glycocalyx and are highly abundant in the nervous tissue. Sialylation is sensed by the innate immune system and acts as an inhibitory immune checkpoint. Aminoglycoside antibiotics such as neomycin have been shown to activate tissue macrophages and induce ototoxicity. In this study, we investigated the systemic subcutaneous application of the human milk oligosaccharide 6’-sialyllactose (6SL) as a potential therapy for neomycin-induced ototoxicity in postnatal mice. Repeated systemic treatment of mice with 6SL ameliorated neomycin-induced hearing loss and attenuated neomycin-triggered macrophage activation in the cochlear spiral ganglion. In addition, 6SL reversed the neomycin-mediated increase in gene transcription of the pro-inflammatory cytokine interleukin-1β (*Il-1b*) and the apoptotic/inflammatory kinase *Pik3cd* in the inner ear. Interestingly, neomycin application also increased the transcription of desialylating enzyme neuraminidase 3 (*Neu3*) in the inner ear. *In vitro*, we confirmed that treatment with 6SL had anti-inflammatory, anti-phagocytic, and neuroprotective effects on cultured lipopolysaccharide-challenged human THP1-macrophages. Thus, our data demonstrated that treatment with 6SL has anti-inflammatory and protective effects against neomycin-mediated macrophage activation and ototoxicity.

## 1 Introduction

Hearing loss is a growing social and economic burden worldwide in an aging population (1, 2). The most common type of hearing loss is sensorineural. Hearing aids and cochlear implants can be used to improve the symptoms and quality of life of patients; however, the loss of auditory sensory neurons and hair cell degeneration remains a chronic, ongoing process. High variability in disease progression has been attributed to genetic susceptibility and environmental factors, such as noise exposure, lifestyle, or diseases (3), which makes the development of a therapy difficult. Acquired sensorineural hearing loss can be caused by aging, drugs, or noise (4). The first signs of age-related acquired hearing loss may already occur at an age of 30 years–39 years in a minority of the population, but the disease is typically slowly progressive, and the prevalence increases to 55% at the age of 70 years–79 years (5). In contrast, drug- and noise-exposure-triggered hearing loss is less frequent but typically follows an acute event that induces acquired hearing loss. We focused on an animal model of aminoglycoside-induced hearing loss with a clear starting event and tested a therapeutic approach that might be relevant for other acquired sensorineural hearing loss events.

Aminoglycosides are important and potent anti-bacterial agents that have been widely used in previous times as antibiotic therapy, despite their severe side effects such as nephrotoxicity and ototoxicity. Although newer and safer antibiotic classes are now used in industrialized countries, aminoglycosides are still in use for local applications or systemically in several underdeveloped countries because of their low costs (6). Aminoglycosides have also been used in several animal models to induce acute hearing loss. Aminoglycoside exposure leads to auditory sensory neurons and hair cell loss in predictable base-to-apex progression, causing deafness (3). Furthermore, treatment with aminoglycosides leads to the activation of macrophages and the production of reactive oxygen species (ROS) in the inner ear of newborn mice (7). Interestingly, the ototoxic effects of neomycin treatment are reported to differ along the tonotopy axis (8).

Sialic acids (Sias) form the terminal ends of the glycocalyx as typical residues in glycoproteins and glycolipids of the cellular surface membrane. Sialylation is highly abundant in the central and peripheral nervous systems. It serves as a checkpoint for the immune system and controls oxidative stress and inflammation (9). Sialylation can be recognized by inhibitory sialic acid-binding immunoglobulin-type lectin (SIGLEC) receptors and complement modulators (e.g., complement factor H and properdin) (9–11), thus controlling innate immune activation. In addition to this anti-inflammatory potential, Sias are known to have anti-oxidative properties owing to their multiple hydroxyl groups. A high local concentration of Sias in the glycocalyx has the potential to scavenge free radical oxygen species (12). During an inflammatory reaction, they can be hydrolyzed by acidic pH, oxidation, or cleaved by cellular neuraminidases.

Milk is rich in Sia residues of oligosaccharides such as sialyllactose. Uptake of these oligosaccharides by breastfeeding helps newborns build up the sialylated glycocalyx of the central nervous tissue during early postnatal development (13). 6’-sialyllactose (6SL), the major form of sialyllactose in humans, possesses non-digestible and therefore non-glycemic properties (14) and can serve as an optimal external Sia source. Studies have shown that over 90% of orally administered sialyllactose can be absorbed in rat pubs, with 30% being retained in the body and 3%–4% in the brain (15). Furthermore, 6SL is tolerated at high concentrations without toxic effects (16), offering a wide therapeutic window. 6SL is a natural milk oligosaccharide that can be synthesized and produced in high amounts. Thus, 6SL is a potent candidate for clinical applications that might act as a source for sialylation to counteract inflammatory processes.

In this study, we investigated the therapeutic properties of human milk oligosaccharide 6SL in an animal model of hearing loss. We treated postnatal mice challenged with neomycin to induce ototoxicity by systemic application of 6SL. We showed that 6SL treatment reversed the neomycin-induced increase in macrophages in the sensory spiral ganglion and partially prevented neomycin-induced hearing loss. We confirmed the anti-inflammatory, anti-phagocytic, and neuroprotective properties of 6SL *in vitro* in a human cell culture model.

## 2 Materials and methods

### 2.1 Experimental animals

All animal experiments were approved by the institutional and governmental review boards of the authors and complied with the Helsinki Declaration. C57BL/6J mice were obtained from the Charles River. All mice were maintained in a specific pathogen-free environment with free access to both water and food and a 12-hour day/night cycle. All efforts were made to minimize the number of animals used and their suffering. For *in vivo* experiments, the day of birth was defined as P0 and breedings were set in-house to obtain 8-day-old mouse pups (P8).

### 2.2 Induction of hearing loss

To induce hearing loss, male and female P8 mouse pubs were injected subcutaneously with either 50 µl 180 mg/kg body weight (bw) neomycin (Sigma, N5285, Germany) in PBS or with 50 µl 180 mg/kg bw neomycin + 60 mg/kg bw of purified 6’-sialyllactose (6SL) in PBS for five consecutive days (P8–P13). This method was modified from that described by Sun et al. (7). As a control, littermates were injected with 50 µl PBS alone. After six days of hearing loss induction, three subcutaneous injections of either 50 µl PBS (for PBS and Neo groups) or 60 mg/kg bw 6SL in PBS were administered (6SL group; P14–P16). The mice were monitored until P28–P30.

### 2.3 Auditory brainstem response test

Auditory Brainstem Response (ABR) test is a minimally invasive approach that measures the electrical activity generated within the auditory nerve and pathways in response to sound (17). We adapted the methods of Rüttiger et al. (18) for this study. In all physiological experiments, the experimenters were blinded to the treatment groups. On days P28–P30, mice were anesthetized and immediately placed in a sound-attenuation chamber on a controlled heating pad (38°C; Fine Science Tools GmbH, Germany) to maintain body temperature. Stainless steel disposable needle electrodes (0.35 mm diameter; Neuro.Dart, SIGMA Medizin Technik GmbH, Germany) were placed subcutaneously at the vertex of the skull and ventral to the pinna of the left ear. The needle electrodes were connected to an extracellular amplifier (WPI DAM-70, World Precision Instruments GmbH, Germany) in the AC power differential mode. A reference needle electrode was subcutaneously placed at the root of the tail. The heart rates of the animals were monitored using electrocardiography throughout the experiment. Auditory stimulation was performed using a speaker (Visaton F 8 SC; driven by a Pioneer A-109 Stereo Amplifier; Pioneer & Onkyo Europe GmbH, Germany) placed 10 cm from the left ear of the animal. The acoustic output of the speaker was measured using a free-field microphone (Bruel & Kjaer Type 4190, Denmark) at the head position of the animal and subsequently compensated in custom recording software to obtain a flat frequency response between 0.5 kHz and 24 kHz up to 100 dB SPL. Acoustic stimuli (100 µs clicks or 1 ms pure tones with 0.2 ms cosine ramps) of alternating polarity were generated at a resolution of 10 µs using custom recording software (MATLAB, R2019a, The MathWorks, Germany) and converted to analog signals (National Instrument PCI-6281, Germany). Each stimulus was presented at least 256 times at a presentation rate of 30 Hz and differential voltage responses (vertex minus ear electrode) were amplified 10,000×, digitized at 10 µs resolution and stored using custom recording software. For analysis, voltage responses were digitally filtered (100 Hz–3,000 Hz band pass filter), and traces containing any signal exceeding 30 µV were rejected as artifacts. The remaining signals were averaged and analyzed between 0 ms and 7 ms post-stimulus onset. Tone or click auditory brainstem response (ABR) thresholds were visually determined by a trained observer blinded to the experimental conditions. The thresholds were objectively validated using the method described by Suthakar and Liberman (19). Furthermore, ABR wave components were algorithmically detected and analyzed for amplitude and delay using a custom software. ABR waveforms were averaged within each treatment group and are presented as the mean wave ± standard deviation.

### 2.4 Tissue collection and preparation

After the ABR test, the mice were directly intracardially perfused with ice-cold PBS while still under deep anesthesia, and the cochleae were immediately dissected on ice as described by Montgomery and Cox (20) for RNA isolation or further immunohistochemistry experiments. For RNA isolation, eight mice per group were used, and for immunohistochemistry, at least five mice per group were analyzed. In summary, the skull was opened quickly and cut in half (sagittal). The forebrain, cerebellum, and brainstem were removed via blunt dissection. Extraneous bones and tissues were carefully dissected from the cochlea, and the cochlea was isolated from the temporal bone. Additional tissues were removed. For immunohistochemistry, the apex was slightly punctured to perfuse the cochlea with 4% ice-cold paraformaldehyde (PFA) via round and oval windows. The cochleae were again post-fixed in 4% PFA overnight, washed for 2 h in PBS, and decalcified in 5% EDTA in PBS while rotating at 4°C. Decalcified cochleae were washed three times in PBS for 15 min and cryoprotected in 30% sucrose (Roth, Karlsruhe, Germany). The left cochlea was then embedded and frozen in O.C.T.^TM^ Compound, Tissue Tek® (Sakura/Fisher Scientific, Schwerte, Germany) and cut into 20 µM sagittal sections using a cryostat Microm Cryo Star HM 560 (Thermo Scientific, Schwerte, Germany). Sections were collected on Superfrost Plus adhesion microscope slides to minimize potential tissue loss during staining (Thermo Scientific, Germany).

### 2.5 RNA isolation and semi-quantitative real-time polymerase chain reaction

For RNA isolation from dissected cochleae, tissue samples were prepared as previously described (21). The tissue was homogenized in 1 ml QIAzol lysis reagent (Qiagen, Germany) with a 5 mm stainless steel bead (Qiagen, Germany) for 5 min at 50 Hz and stored at −80°C until use. For RNA isolation from cells, cultures were washed with PBS and scraped in 1 ml QIAzol Lysis reagent (Qiagen, Germany) for further use. RNA was then extracted similarly for tissue and cell samples by first incubating for 5 min at RT and then adding 200 µM chloroform (Roth, Germany). After incubation for 3 min at room temperature, samples were centrifuged at 12,000 rpm for 15 min at 4°C. The upper phase, containing the RNA, was transferred to a new tube. Isopropanol (Roth, Germany) was added in a 1:1 ratio, and the samples were vortexed well and incubated overnight at −20°C. The following day, the samples were centrifuged again at 12,000 rpm for 20 min at 4°C. The supernatant was discarded, and the samples were washed three times by adding 300 µl of 70% ethanol (Roth, Germany) in PBS, centrifuged at 12,000 rpm for 5 min at 4°C, and the supernatant was discarded. After the last wash, the supernatant was discarded, and the samples were left to dry for 10 min–20 min at room temperature. RNA was resuspended in 11 µl RNAse-free DEPC water (Invitrogen, USA). RNA concentration was measured using a Nanodrop spectrometer (Thermo Fisher Scientific, USA). Reverse transcription of RNA was performed using SuperScript III reverse transcriptase and hexamer random primers (both from Invitrogen, Germany) according to the manufacturer’s protocol for SuperScript First-Strand Synthesis. Semi-quantitative real-time polymerase chain reaction (sqRT-PCR) with specific oligonucleotides (**Supplementary Tables 3, 4**) was performed according to the manufacturer’s protocol using SYBR Green PCR Master Mix (Invitrogen, Germany) and Mastercycler epigradient S (Eppendorf, Wesseling-Berzdorf, Germany). Transcripts of the housekeeping gene *glyceraldehyde-3-phosphate dehydrogenase (GAPDH)* were used as internal RNA loading controls. Amplification specificity was confirmed by melting curve analysis. The results were analyzed using Mastercycler ep realplex software (Eppendorf, Germany) after establishing the reaction efficiency for each primer pair. The values were normalized to their respective *GAPDH* values and quantified using the delta delta-CT method. For each animal, the mean CT read-out value for *GAPDH* was calculated and subtracted from the mean CT value of each gene primer of interest, yielding the delta CT value. The mean delta CT value for the PBS control group was calculated. The delta delta-CT value of each gene primer for each animal was then calculated by subtracting the mean delta CT value of the PBS control group from the delta CT value of the respective primer. The power of the negative delta delta-CT value for each gene primer for each animal was then calculated and used as the relative read-out value for each gene transcription.

### 2.6 Immunohistochemistry of cochlea tissue

For neuronal cell density analysis, cryosections were washed with PBS and incubated in a blocking solution (10% BSA + 0.2% Triton X-100 + 5% NGS) for 1 h at room temperature. Primary antibodies for neuronal nuclei (NeuN; 1:500; Millipore #MAB377, Germany) diluted in blocking solution were added and the sections were incubated for 2 h at room temperature. After washing the sections three times with saline, the secondary antibody Alexa Fluor 647 (1:250; Dianova #115-606-072, Germany) diluted in blocking solution was added and incubated for another 2 h at room temperature. The sections were washed again twice and stained with DAPI (1:10,000, Sigma, Germany) before mounting with Aqua-Polymount (Polysciences Inc., Germany). Images of the spiral ganglion were taken using a magnification of ×40 and at 2 µM intervals over five optical sections with an SP8 confocal microscope and LAS-X software (Leica, Wetzlar, Germany). For the quantification of macrophages, cryosections were washed initially with PBS and incubated in blocking solution (10% BSA + 0.2% Triton X-100 + 5% NGS in PBS) for 2 h at room temperature, followed by incubation in primary antibodies directed against Iba1 (1:500; IBA1 rabbit-anti, Wako #019-19741, Germany) and against Cd68 (1:500, rat-anti-mouse, AbD Serotec, MCA1957, UK) in blocking solution overnight at 4°C. After thorough washing with saline, the sections were stained with the corresponding secondary antibodies (Alexa Fluor^®^ 647-conjugated goat anti-rabbit IgG, 1:500, Dianova #111-606-144, Germany and Cy3 goat anti-rat, 1:200, Dianova #112-166-072; Germany) in PBS for 2 h at room temperature. After washing with saline, the sections were stained for nuclei (DAPI, 1:10,000) for 30 s and then covered with Aqua-Polymount (Polysciences Inc.). Images of the spiral ganglion were taken at a magnification of ×40 and at 2 µM intervals over five optical sections using an SP8 confocal microscope and LAS-X software (Leica, Wetzlar, Germany).

### 2.7 Morphological analysis of macrophages

The maximum projections of the Iba1-/DAPI-double-positive z-stack images were generated and analyzed with ImageJ (v.1.52u) using custom-written plugins. Macrophage density was quantified by counting the Iba1-/DAPI-double-positive cells per area in each image and calculating the average for each mouse. To determine the overall Iba1 expression, the total fluorescence integrated density was measured for each image and analyzed by subtracting the background fluorescence integrated density measured in the primary antibody negative control. The mean fluorescence intensity was calculated per mouse, and the mean for each experimental group was calculated and normalized to that of the control group.

### 2.8 Purification of sialyllactose

6’-sialyllactose (6SL) purchased from Carbosynth was further purified using an FPLC approach with a HiPrep Q XL 16/10 column. The HiPrep Q XL 16/10 column was washed with Ampuwa water before loading it with 50 mg of 6SL (Carboysynth) dissolved in 1 mL of Ampuwa water. The column was washed with Ampuwa water to remove impurities, followed by a linear gradient of 1 M ammonium bicarbonate to elute the 6SL. The eluate was collected, frozen to −80°C and freeze-dried using the ALPHA 1-2/LD Plus freeze dryer at a pressure of 0.1 mbar for 24 h. Lyophilized 6SL was then reconstituted under sterile conditions, and its concentration was determined using the Aminoff method (22).

### 2.9 Human THP1 macrophage cell culture

Human monocytes (THP1 cells, kindly provided by Prof. Hornung, Bonn, Germany) were cultured in RPMI medium supplemented with 1% heat-inactivated chicken serum (Gibco, Germany), 1% penicillin/streptomycin (Gibco, Germany), 1% L-glutamine (Gibco, Germany), 1% sodium pyruvate (Gibco, Germany), and 1% N2 (Gibco, Germany). For differentiation, floating THP1 monocytes were plated and incubated with 10 ng/ml Phorbol-12-Myristate-13-Acetate (PMA; Sigma, Germany) in a prewarmed medium for 48 h to allow adherence. Cells were washed three times with prewarmed medium to remove the remaining PMA and were incubated for 48 h at 37°C and 5% CO2. After 18 h, the cells showed macrophage-like morphology and could be used for experiments (further described as macrophages).

### 2.10 Co-culture of human sensory neurons and human THP1 macrophages

Human sensory neurons were obtained from human induced pluripotent stem (iPS; WiCell foreskin-1) cells, as described by Chambers et al. (23). On day 0, the iPS colonies were detached by collagenase IV (Gibco, Germany), collected by sedimentation, and resuspended in DMEM/F12 medium (Gibco, Germany) supplemented by 20% knockout serum replacement (Gibco, Germany), 1% nonessential amino acids (Gibco, Germany), 10 µM SB431542 (inhibitor of transforming growth factor b1 and activin receptors; Axom Medchem, Netherlands), 1 µM dorsomorphin (Sigma, Germany), 3 µM CHIR99021 (inhibitor of glycogen synthase kinase [GSK] 3b and GSK-3a; Axom Medchem, Netherlands), and 0.5 µM purmorphamine (Sigma, Germany) for 2 days. On day 2, the medium was replaced by 50:50 DMEM/F12: neurobasal, 1:200 N2 supplement (Gibco, Germany), 1:100 B27 supplement (N2B27 medium, Gibco, Germany) supplemented with 10 µM SB431542, 1 µM dorsomorphin, 3 µM CHIR, and 0.5 µM purmorphamine for 2 days. On day 4, 150 µM ascorbic acid (Tocris, United Kingdom) was added to the N2B27 medium, but SB431542 and dorsomorphin were withdrawn. On day 6, embryonic bodies were triturated and seeded on plates coated with Geltrex (Thermo Fisher Scientific, Germany) at low density and expanded in N2B27 medium supplemented with CHIR99021, ascorbic acid, and purmorphamin for five passages. The neural stem cells (NSCs) were then ready for differentiation. For differentiation, NSCs were split using accutase (Sigma, Germany) and replated in Geltrex-coated plates. NSCs were treated for 2 days with N2B27 medium supplemented with 3 µM CHIR99021 and afterwards for one week with 10 ng/ml BMP4 (Novus Biologicals, Germany). Consequently, the premature neurons were split by accutase and replated at a density of 20,000 cells per well in Geltrex-coated chamber slides in N2B27 maturation medium containing 10 ng/ml brain-derived neurotrophic factor (BDNF; Prospect, Israel), 10 ng/ml glial cell line-derived neurotrophic factor (GDNF; Prospect, Israel), and 500 µM dbcAMP (Sigma, Germany) for 2 weeks. In parallel, the human THP1 monocyte cell line was maintained in RPMI medium, as described above. Cells were incubated with PMA for 48 h, washed twice with the medium, and then incubated for an additional 48 h without PMA. For the experiments, THP-1 macrophages were scraped, counted, and added to the sensory iPS cell-derived neurons in a 1:1 ratio of co-culture medium (N2B27 medium plus BDNF, GDNF, and dbcAMP) and treated with 100 µM 6SL or vehicle control for another 48 h.

### 2.11 Neurite lengths analysis after co-culture with macrophages

Human sensory neuron-macrophage co-cultures were washed once with 1× PBS and fixed with 4% paraformaldehyde (PFA) for 15 min at room temperature (RT). Then, non-specific binding sites of the fixed co-culture were blocked, and the co-culture was stained with antibodies against rabbit anti-neurofilament (1:1,000; Sigma, USA) and rat anti-CD11b (2 µg/mL; BD Biosciences #553307, Germany) overnight at 4°C. The following day, the fixed co-cultures were washed three times with blocking solution and incubated with secondary antibodies (1:500 Alexa488-conjugated anti-rabbit antibody, #A11008 Invitrogen, Germany and 1:500 Cy3-conjugated anti-rat antibody, #112-166-072 Dianova, Germany) for 2 h at RT. Cells were washed three times with 1× PBS and subsequently labeled with 4’,6-diamidino-2-phenylindole (DAPI, 1:5,000, Sigma). Images were captured using a confocal laser-scanning microscope (Fluoview 1000, Olympus). For quantification of neurite length, a total of 10 pictures per condition were collected, maintaining the same settings, and analyzed using ImageJ/NeuronJ software (NIH, MBF) by a blinded investigator. Images were processed equally, and the mean length of the neurofilament-positive neurites was quantified. The mean length of the untreated control cells was set to 100%.

### 2.12 Bead phagocytosis of THP1

Human macrophages (THP-1) were seeded at 96 h (48 h with and 48 h without PMA) prior to stimulation as described above. The cells were stimulated with 3 µg/ml LPS (InvivoGen) and different concentrations of 6SL for 24 h. After 23.5 h Fluoresbrite® Polychromatic Red Microspheres 1.0 μm beads (Polysciences Inc., USA) were added and incubated for 30 min. The cells were then washed and collected in PBS for analysis using flow cytometry (Accuri; BD, Germany). The percentage of cells that took up two or more beads was determined and normalized to the untreated condition (UT). The untreated condition was set as 100%.

### 2.13 Radical oxygen species analysis

THP1 macrophages were cultured and seeded as described above (48 h with and 48 h without PMA). Cells were pre-incubated with 6SL (5 µM), SOD (60 U/ml; Sigma, Germany), or Trolox (40 µM; Merck, Germany) for 1 h before stimulation with LPS (3 µg/ml, InvivoGen, Germany) for 15 min at 37°C. Cells were washed and incubated in 30 µM DHE in Krebs-HEPES buffer for another 15 min at 37°C under dark light conditions. The cells were then washed three times in Krebs–Hepes buffer, scraped, centrifuged, and resuspended in PBS for direct flow cytometry analysis (Accuri; BD, Germany). The mean DHE intensity of the flow cytometry analysis was normalized to that of the untreated condition (UT). The untreated condition was set to 1.0.

### 2.14 Ethics

All animal experiments were conducted according to the authors’ institutional guidelines by the local government and the principles expressed in the Declaration of Helsinki. Experiments were conducted in accordance with the European Directive (2010/63/EU) on the protection of animals used for experimental and other scientific purposes. The Animal Ethics Committee of the University of Bonn and the local government approved all procedures.

### 2.15 Statistical analysis

Data with two experimental groups were tested for normality and then analyzed by either Student’s t-test or Mann–Whitney test, or if group number >2 by one-way ANOVA followed by Bonferroni *post hoc* test or Dunnett’s T3 method comparing all columns using the IBM SPSS Statistics (v.22; IBM Corporation, Germany). Data were considered significantly different if *p ≤0.050, **p ≤0.010, or ***p ≤0.001. The absence of statistical notation indicates that there were no statistically different outcomes. Click ABR thresholds, wave I metrics per sound pressure level, and immunohistochemistry staining analysis were compared among the three treatment groups using one-way ANOVA. For tone ABR thresholds, two-way ANOVA (treatment × frequency) was used. For all *post-hoc* pairwise t-tests, the significance levels were adjusted according to Tukey’s honestly significant difference (HSD) procedure (MATLAB multcompare.m).

## 3 Results

### 3.1 6’-sialyllactose partially rescued the neomycin-induced hearing impairment in auditory brainstem responses

**Figure 1:**
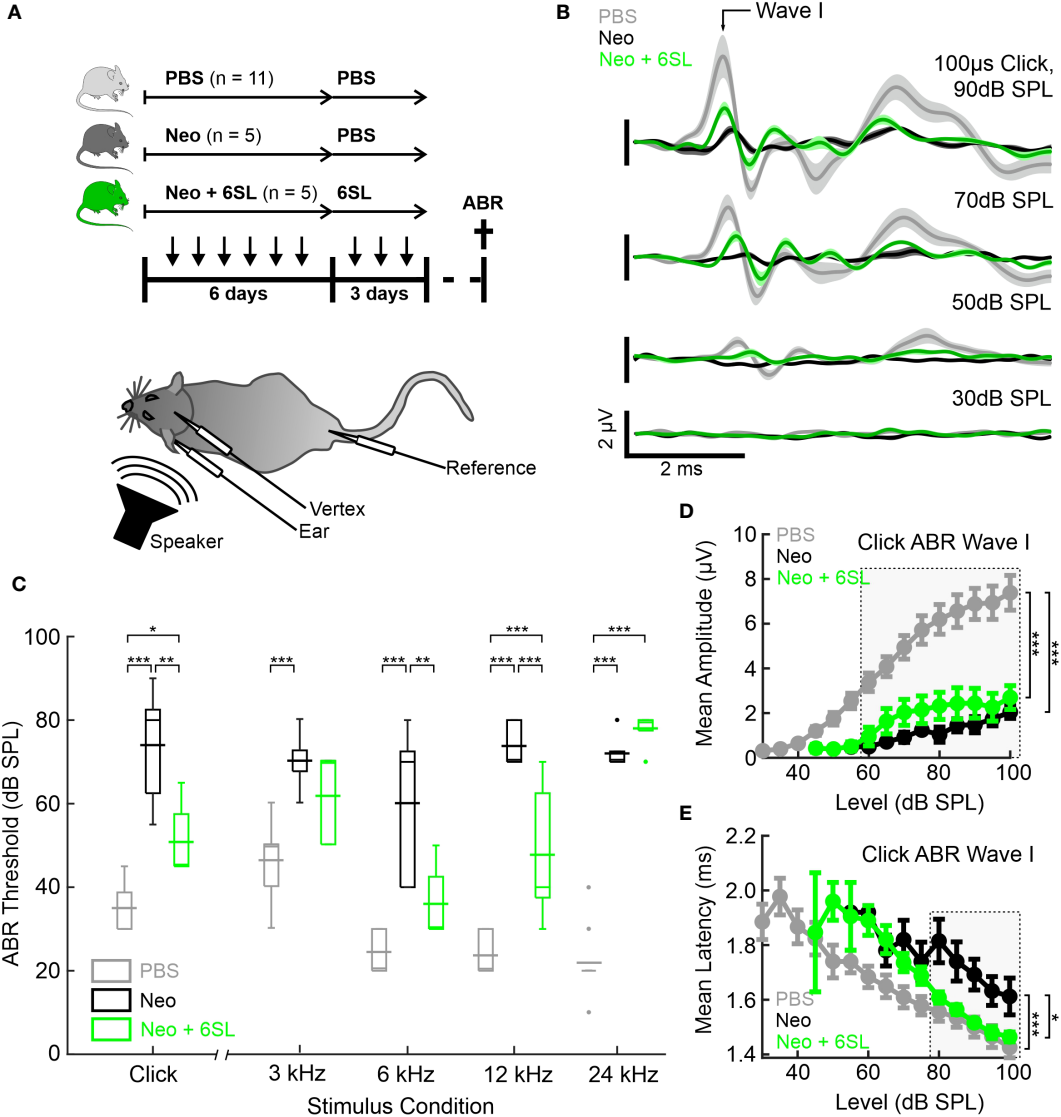
Systemic application of 6’-sialyllactose ameliorated the neomycin-induced hearing loss. **(A)** Auditory brain responses (ABR) were measured with electrodes (vertex, ear and reference) after noise exposure (speaker) in three experimental groups, namely control animals (vehicle control PBS, light gray), neomycin-treated animals (Neo, dark gray), and neomycin plus 6SL treated animals (Neo + 6SL, green). Mice received six repeated daily applications of either Neo, Neo + 6SL, or saline control, and subsequent repeated daily applications of 6SL or saline control, as indicated by the arrows on the timeline. Mice were sacrificed on days P28–P30 (cross on the timeline) after being subjected to ABR recordings. A schematic drawing of the mouse shows the electrode and speaker placement positions relative to the animal. **(B)** Click ABR waveforms at four sound pressure levels (30 dB, 50 dB, 70 dB, and 90 dB) are shown as mean (solid lines; n = 11/5/5 for PBS, Neo, and Neo + 6SL) ± standard deviation (shaded areas). Treatment with 6SL partially rescued the wave lost after neomycin application. One set of representative recordings was presented. **(C)** Box plots of auditory thresholds measured by ABR for different stimulus conditions. The auditory threshold increased in the Neo group and ameliorated in the Neo + 6SL group under distinct stimulus conditions. Thick horizontal lines crossing the boxplot indicate mean values and thin lines in the boxplots indicate median values. Asterisks indicate statistically significant differences between groups, marked by brackets: *p <0.05, **p <0.01, ***p <0.001. The absence of statistical notation indicates that there was no statistically significant difference. **(D, E)** Analysis of the click-evoked ABR Wave I component, shown as mean ± standard deviation amplitudes **(D)** and mean ± standard deviation peak latencies **(E)** vs. click sound pressure levels between 30 dB SPL and 100 dB SPL. At higher sound pressure levels (SPL), 6SL ameliorated the neomycin-induced increase in the ABR Wave I latency **(E)**. *p <0.05, **p <0.01, ***p <0.001 ANOVA. Brackets refer to the 90 dB SPL condition.

Aminoglycoside antibiotics, including neomycin, are ototoxic to both humans and mice. Neomycin has been applied repeatedly in postnatal mice to induce hearing loss associated with local activation of macrophages (7). Therefore, we performed repeated daily applications of neomycin and tested the therapeutic potential of the co-application of 6SL on auditory brainstem responses (ABR) in early postnatal mice (**Figure 1A**). For this therapeutic approach, bovine milk-derived 6SL was further purified prior to experimentation with a fast protein liquid chromatography (FPLC)-based anion-exchange chromatography (AEX)-chromatography approach to remove residual contaminants. Mice were injected daily for 6 days starting at postnatal age P8 with either vehicle as control (PBS), 180 mg/kg bw neomycin (Neo), or 180 mg/kg bw neomycin plus 60 mg/kg bw 6SL (Neo + 6SL). Subsequently, the respective animal groups received daily injections of PBS (PBS group and Neo group) or 6SL (Neo + 6SL group) over three additional days (**Figure 1A**). After 12 days–14 days the animals were subjected to electrophysiological ABR measurements and immediately analyzed using immunohistochemistry. No significant difference in body weight was observed among the distinct experimental groups at the day of analysis 13.5 g± 3.1 g (PBS group), 15.0 g ± 0.6 g (Neo group), 13.9 g ± 1.1 g (Neo + 6SL group) (see **Supplementary Table 1**).

Typical ABR waveforms were obtained from mice in all experimental groups (**Figure 1B**). In neomycin-treated mice (Neo group), ABR waveforms were only observed at higher sound pressure levels (SPL; 70 dB and 90 dB) and showed reduced ABR amplitudes and delayed wave components even at 90 dB SPL compared to the PBS group. For the Neo + 6SL treated animals, this was to a certain extent also the case with a reduction in amplitude, but ABR waveforms were still visible at higher sound pressure levels. Therefore, we quantified the ABR thresholds of animals in the three treatment groups (**Figure 1C**). Control animals treated with vehicle only (PBS) had a click ABR threshold of 35 dB ± 6 dB SPL. Neomycin treatment caused profoundly elevated click ABR thresholds (74 dB ± 14 dB SPL; p <0.001) in mice. Animals treated with Neo + 6SL showed reduced click ABR thresholds, from 74 dB ± 14 dB to 51 dB ± 9 dB SPL (p = 0.002, ANOVA *Post-hoc* pairwise testing Tukey’s HSD). Thus, treatment with 6SL led to a significant reduction in click ABR thresholds (ANOVA F = 32.8, total df = 20, p <0.001).

Since ototoxic effects of neomycin treatment are reported to differ along the axis of tonotopy (8), we next measured tone ABR thresholds in the three experimental groups (**Figure 1C**). Control mice (PBS) showed a normal audiogram (3 kHz: 46 dB ± 9 dB SPL; 6 kHz: 25 dB ± 5 dB SPL; 12 kHz: 24 dB ± 5 dB SPL; and 24 kHz: 22 dB ± 8 dB SPL). In the Neo-treated mice, tone ABR thresholds were elevated (3 kHz: 70 dB ± 7 dB SPL; 6 kHz: 60 dB ± 19 dB SPL; 12 kHz: 74 dB ± 5 dB SPL; 24 kHz: 72 dB ± 4 dB SPL), with the most severe effects evident for higher frequencies, where an average threshold elevation of +50 dB SPL was found. In the Neo + 6SL group, a mixed picture emerged: Tone ABR thresholds for frequencies of 12 kHz and below were less increased compared to the Neo group (3 kHz: 62 dB ± 11 dB SPL; 6 kHz: 36 dB ±9 dB SPL; 12 kHz: 48 dB ± 16 dB SPL; 24 kHz: 78 dB ± 5 dB SPL), while the tone ABR threshold for 24 kHz was severely elevated. Statistical analysis using two-way ANOVA revealed that both frequency (F = 17.9, df = 83, p <0.001) and treatment (F = 158, df = 83, p <0.001) had significant effects on tone ABR thresholds. Furthermore, a significant interaction (F = 11.2, df = 83, p <0.001) between the frequency and treatment groups supported the finding that both the outcome of neomycin treatment and rescue by 6SL differed depending on the tonotopic position. *Post-hoc* pairwise testing (Tukey’s HSD; **Supplementary Table 2**) supported this conclusion. The most severe neomycin effects in the basal high-frequency region (24 kHz condition) were unaffected by 6SL but were rescued in the middle frequency regions (6 kHz and 12 kHz) (**Supplementary Table 2**). Thus, treatment with 6SL decreased the tone ABR thresholds compared to the Neo group, showing a significant amelioration of the neomycin-induced damage by 6SL in middle-frequency regions of the cochlea.

To better understand the outcome of the effect of 6SL, we next analyzed the peak amplitude (**Figure 1D**) and latency (**Figure 1E**) of the ABR wave I component in click-evoked ABR at varying sound pressure levels. Average wave I amplitudes in control animals (PBS) grew from the threshold with a sigmoidal function for the control (PBS) and 6SL-treated groups (Neo + 6SL), whereas the growth function of ABR wave I amplitude was much shallower in neomycin-treated animals. Wave I amplitude was affected by both the Neo and Neo + 6SL groups for all sound pressure levels above 55 dB SPL (ANOVA 55 dB SPL: F = 7.4, df = 15, p = 0.007). At high sound pressure levels (ANOVA 90 dB SPL: F = 20.1, df = 20, p <0.001), the PBS group differed from both the Neo (p <0.001) and 6SL (p <0.001) groups in *post-hoc* pairwise tests (Tukey’s HSD). Thus, the amplitude of the ABR wave component generated by hair cells was not restored to normal levels by 6SL treatment. This finding could be explained by either a reduced number of the generators or by an uncoherent activation of generators of the ABR wave component. Therefore, we also analyzed ABR wave I latencies (**Figure 1E**). At high sound pressure levels, Wave I latencies were lower in the Neo + 6SL group than in the Neo group (p = 0.035, ANOVA *post-hoc* pairwise test). Thus, analysis of ABR Wave I amplitude suggests that in both the Neo and Neo + 6SL rescue conditions, the number of functional ABR generators was reduced compared to the PBS control group. Only for the ABR Wave I latency was a reduction observed at higher SPL in the Neo + 6SL group than in the Neo group.

In summary, the data showed that 6SL treatment rescued neomycin-induced hearing loss in mice, particularly in the middle frequency regions, and ameliorated the click ABR threshold.

### 3.2 6’-sialyllactose prevented the strong increase in number of macrophages in the cochlear spiral ganglion

**Figure 2:**
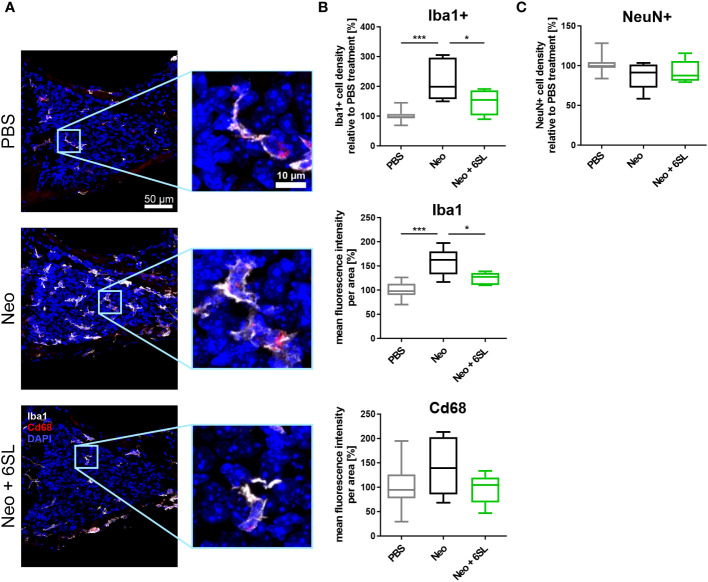
Treatment with 6’-sialyllactose prevented the increase in number of macrophages in the cochlear spiral ganglion. **(A)** Cryosections of the cochlear spiral ganglion were stained with an antibody against the macrophage marker ionized calcium binding adaptor molecule 1 (Iba1; white), lysosome-related protein Cd68 (red), and nuclear marker 4′,6-diamidino-2-phenylindole (DAPI, blue). An increased number of macrophages was visible in the neomycin-treated spiral ganglia compared to that in the PBS- and Neo + 6SL-treated groups. Representative z-stack images of the cochlear spiral ganglion of mice treated with PBS, neomycin (Neo), or neomycin with 6’-siallylactose (Neo + 6SL) are shown. Scale bar: 50 µM; higher magnification: 10 µM. **(B)** The number of Iba1-positive macrophages and intensities of Iba1 and Cd68 were quantified in the cochlear spiral ganglia. Quantification of Iba1-positive cell density revealed significantly more macrophages in the Neo group than in the PBS or Neo + 6SL groups. The Iba1 fluorescence intensity was strongly upregulated after neomycin treatment compared to that in the PBS group. This upregulation was attenuated after simultaneous 6SL treatment of the neomycin-challenged mice. The Cd68 intensity showed no change among the groups. Data are shown as a min to max box + whisker plot; n = 5–11. **(C)** Quantification of the relative cell density of neuronal nuclei (NeuN)-positive cells in cochlear spiral ganglia. No difference was observed; however, the number of NeuN-positive cells was slightly decreased in the Neo group. Data shown as min to max box + whiskers plot; n = 5–11. All data were analyzed by one-way ANOVA and Tukey’s multiple comparison test, *p <0.05, ***p <0.001.

To understand what happened at the cellular level, we examined the macrophages in the inner ear. Neomycin treatment can increase the number and/or activation of macrophages in the inner ear (7). Therefore, we analyzed the number and expression levels of the macrophage marker Iba1 in the cochlear spiral ganglion, where the cell bodies of bipolar neurons innervate the hair cells (**Figure 2A**). We found only a low number of Iba1+ macrophages in the spiral cochlear ganglion of the PBS control group. Challenge of mice with neomycin increased the number of Iba1+ cells in the cochlear spiral ganglia, an effect that was reversed in the Neo + 6SL group (**Figure 2A**). We quantified the number and intensity of Iba1 staining (**Figure 2B**). Neomycin treatment increased the relative number of Iba1+ macrophages from 100% ± 18.3% to 221% ± 71.3% (p <0.001) (**Figure 2B**). This increase in Iba1+ macrophages was reversed from 221% ± 71.3% to 147% ± 43.3% (p = 0.028, Neo vs. Neo + 6SL group) after treatment with 6SL.

We also quantified the overall intensity of Iba1 and lysosomal activation marker Cd68 in the spiral ganglion. The overall intensity of Iba1 per area increased in the Neo group (from 100% ± 17.1% in PBS to 158% ± 29.1% in the Neo group; p ≤0.001), which was reversed in the Neo + 6SL group (124% ± 12.8%; p = 0.038 vs. Neo group), confirming the increased amount or activation of macrophages in the cochlear spiral ganglia (**Figure 2B**). The Cd68 intensity per unit area did not change among the groups.

Next, we analyzed neuronal density in the spiral ganglion by staining with the neuronal nuclei marker (NeuN). No significant difference in neuronal density between the three experimental groups was visible, although slightly fewer neurons were observed in the Neo group (PBS: 101% ± 10.6%; Neo: 87.8% ± 17.7%; 92.2% ± 14.4%, **Figure 2C**).

Thus, the data showed an increased number of Iba1+ macrophages as a sign of immune system activation in the spiral ganglia after treatment with Neo, which was reversed by the application of 6SL.

### 3.3 6’sialyllactose ameliorated the neomycin-induced increase of the inflammation-related *Aif1* and *Pik3cd* gene transcription

**Figure 3:**
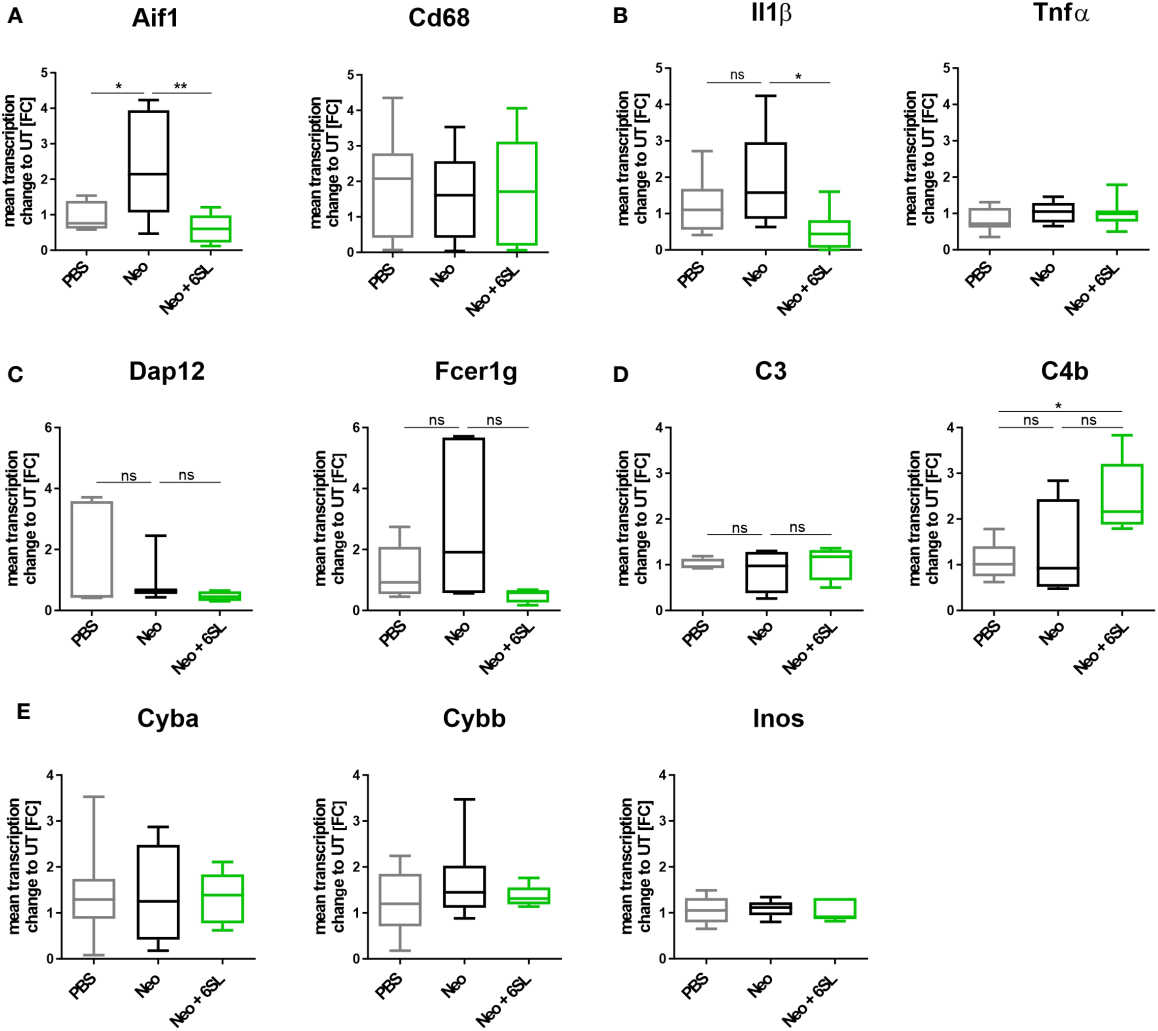
Treatment with 6’-sialyllactose ameliorated the neomycin-induced increase of immune-related gene transcription. **(A)** Transcription levels of allograft inflammatory factor 1 (*Aif1*; gene of Iba1) and the CD68 antigen (*Cd68*) of whole cochlea homogenates from mice treated with PBS, neomycin (Neo), or neomycin with 6’-siallylactose (Neo + 6SL). The *Aif1* transcript levels were higher in the Neo group than in the PBS control group. This increase was not observed in the Neo + 6SL group. The *Aif1* transcript levels decreased after additional treatment with 6SL. *Cd68* transcript levels remained unchanged across all treatment groups. Data are shown as a min to max box + whisker plot; n = 7–8. **(B)** 6SL decreased the transcription of the proinflammatory cytokine interleukin 1β (*Il1b*) in whole cochlear homogenates from mice treated with 6SL (Neo + 6SL) compared to that in neomycin (Neo) treatment group. Transcription of tumor necrosis factor-α (*Tnfα*) did not change. Data are shown as a min to max box + whisker plot; n = 6–8. **(C)** Gene transcription of the tyrosine kinase-binding protein (*Tyrobp/Dap12*) was unchanged in the neomycin (Neo) and neomycin groups treated with 6’-siallylactose (Neo + 6SL) compared to the control group (PBS). Likewise, gene transcription of the phagocytic marker Fc fragment of IgE, high affinity I receptor for gamma polypeptide (*Fcer1g*), was unchanged in the Neo and the Neo + 6SL groups compared to the control group. Data are shown as a min to max box + whisker plot; n = 3–5. **(D)** Gene transcription of complement marker C3 (*C3*) was unchanged in the neomycin (Neo) and neomycin groups treated with 6’-siallylactose (Neo + 6SL) compared to the control group (PBS). Transcription levels of complement marker C4b (*C4b*) were higher in the Neo + 6SL group than in the PBS control group. Data are shown as a min to max box + whisker plot; n = 4–5. **(E)** Transcription levels of *Cyba*, *Cybb*, and *Inos/Nos2* were analyzed in whole cochlear homogenates for the oxidative burst pathway. Neither neomycin nor 6SL treatment had any effect on these gene transcripts compared with the PBS control group. Data are shown as a min to max box + whisker plot; n = 5–7. All data were analyzed by one-way ANOVA, Tukey’s multiple comparison test, *p <0.05, **p <0.01, ns, not significant.

To examine the increased immune activation at the gene transcription level, we analyzed gene transcripts of pro-inflammatory, oxidative, and apoptotic/necroptotic pathways in the whole inner ear. We found elevated transcription levels of the macrophage marker *Aif1 (Iba1)* in the Neo-treated compared to the PBS control group (**Figure 3A**). Specifically, *Aif1* gene transcription was increased from 0.927 ± 0.4-fold change (FC) to 2.37 ± 1.5 FC (p = 0.026), which was reversed by 6SL to 0.664 ± 0.4 FC (p = 0.008). Transcription of the macrophage lysosomal marker *Cd68* was not affected by neomycin or 6SL. Next, we analyzed the proinflammatory cytokine gene transcripts of interleukin 1β (*Il1b*) and tumor necrosis factor-α (*Tnfα*). The transcript levels of *Il1β* were higher in the Neo group than in the Neo + 6SL group (**Figure 3B**). In detail, gene transcription of *Il1b* was increased from 1.22 ± 0.8 FC to 1.92 ± 1.36 FC (p = 0.366) by treatment with Neo, an effect that was reverted in the Neo + 6SL group (0.56 ± 0.2 FC, p = 0.043). In contrast, *Tnfα* transcript levels showed no change. We further checked for gene transcription of phagocytic markers, such as the DNAX activating protein 12 kDa (*Dap12*) and the immunoreceptor tyrosine-based activation motif (ITAM)-signaling Fc fragment of IgE, high affinity I receptor for gamma polypeptide (*Fcer1g*). For both genes, no changes were observed between the groups (**Figure 3C**). Since it is known that the complement system is involved in certain phagocytic processes led by macrophages, we also examined two key complement factors. While the transcription of complement factor *C3* was not affected by neomycin treatment, we observed an upregulation in *C4b* transcript levels after Neo treatment, which was elevated in the Neo + 6SL group compared to that in the PBS control group (**Figure 3D**). To understand the mechanistic link between immune activation and toxicity, we checked the transcription levels of markers of oxidative pathways. Neither the transcription levels of *Cyba* and *Cybb*, the genes encoding for the subunits of NADPH oxidase 2 (nicotinamide adenine dinucleotide phosphate oxidase) or *iNos/Nos2*, were changed in the whole inner ear two weeks after the end of treatment (**Figure 3E**).

**Figure 4:**
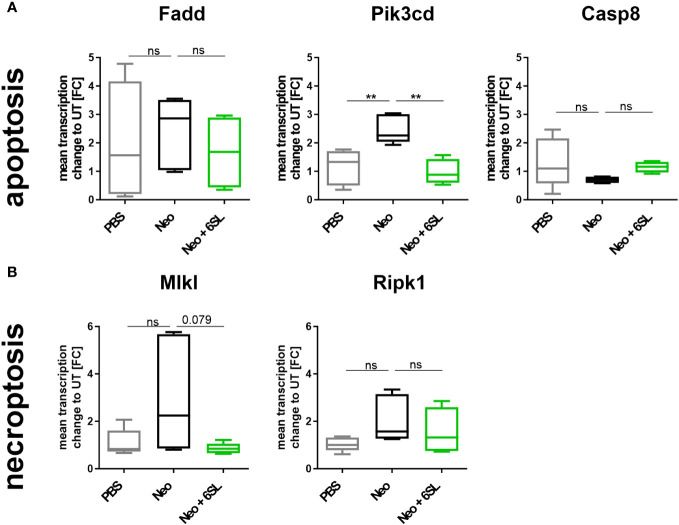
Effects of neomycin and 6’-sialyllactose on the apoptotic and necroptotic pathway-related gene transcription. **(A)** Transcription of the apoptosis pathway-related genes associated with the death domain (*Fadd*), phosphatidylinositol-4,5-bisphosphate 3-kinase catalytic subunit delta (*Pik3cd*), and caspase 8 (*Casp8*) were analyzed. Only the apoptosis/inflammation-related *Pik3cd* was elevated in whole cochlea homogenates of neomycin-treated mice (Neo) compared to the PBS control group and was reversed after additional treatment with 6’-siallylactose (Neo + 6SL). Data are shown as a min to max box + whisker plot; n = 4–6. **(B)** Transcription of the necroptosis-related genes mixed lineage kinase domain like pseudokinase (*Mlkl*) and receptor-interacting serine/threonine-protein kinase 1 (*Ripk1*) showed no difference among the groups, although *Mlkl* tended to decrease in the Neo + 6SL group compared to the Neo group. Data are shown as a min to max box + whisker plot; n = 4–6. All data were analyzed by one-way ANOVA, Tukey’s multiple comparison test, **p <0.01, ns, not significant.

Reduced hearing ability could be a result of an increased number of dying cells in the inner ear. Although morphologically dying cells are difficult to detect, we wondered whether certain markers were increased after neomycin treatment. Therefore, we examined the gene transcripts involved in apoptosis and necroptosis by analyzing the transcription of the apoptosis marker fas associated via death domain (*Fadd*), phosphatidylinositol-4,5-bisphosphate 3-kinase catalytic subunit delta (*Pik3cd*), and caspase 8 (*Casp8*) (**Figure 4A**), and the necroptosis markers mixed lineage kinase domain like pseudokinase (*Mlkl*) and receptor-interacting serine/threonine-protein kinase 1 (*Ripk1*) (**Figure 4B**), in the whole inner ear. *Pik3cd* showed an upregulation after neomycin treatment compared to the PBS group (from 1.17 ± 0.6 FC to 2.47 ± 0.5 FC, p = 0.004) that was completely prevented in the therapy Neo + 6SL group (0.988 ± 0.4 FC, p = 0.002). Interestingly, gene transcript of the necroptotic pathway for *Mlkl* (1.1 ± 0.5 FC to 2.93 ± 2.35 FC, p = 0.108) showed a tendency for decreased levels in the Neo + 6SL group compared to the Neo group, (0.85 ± 0.2 FC, p = 0.079; **Figure 4B**).

In summary, the data showed upregulation of *Aif1* and *Pik3cd* gene transcription in the inner ear in the Neo group, which was reversed in the Neo + 6SL therapy group.

### 3.4 Neomycin- and 6'-sialyllactose-induced changes in sialylation-related gene transcripts

**Figure 5:**
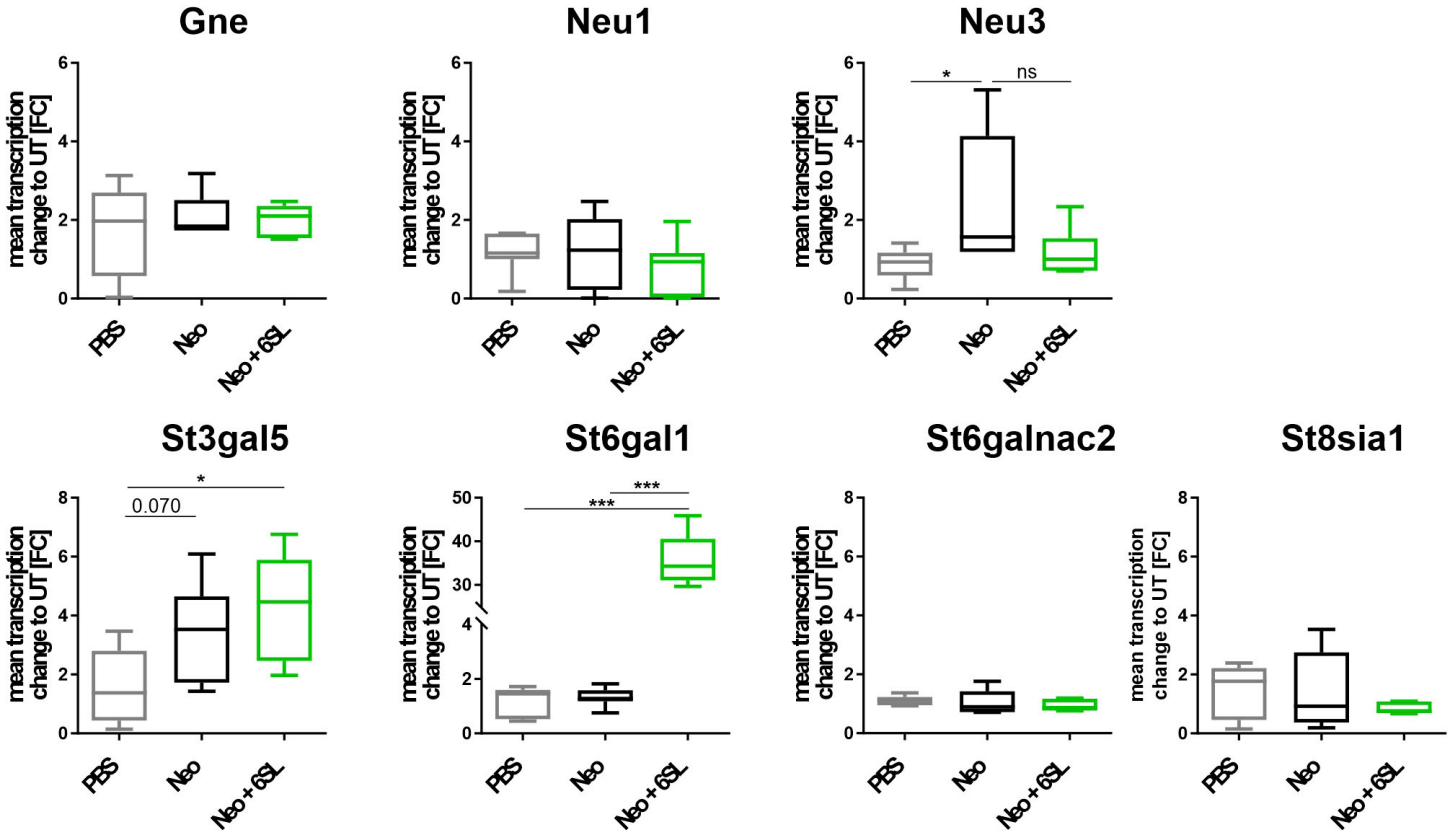
Neomycin and 6SL-induced changes of the sialylation-related genes transcript. The transcription levels of different genes in the sialylation regulation pathway were analyzed by qRT-PCR. Gene transcription of glucosamine-2-epimerase/N-acetylmannosamine kinase (*Gne*) and neuraminidase 1 (*Neu1*) in the cochlea were not affected by neomycin treatment, whereas the transcription level of neuraminidase 3 (*Neu3*) was upregulated in the neomycin group (Neo) compared to the PBS group. Gene transcription of the sialyltransferases *St3gal5* and *St6gal1* was upregulated in the Neo + 6SL group compared to that in the PBS control group. Transcription levels were not altered in the sialyltransferase *St6galnac* or polysialyltransferase *St8sia1*. Data are shown as a min to max box + whisker plot; n = 5–8. All data were analyzed using one-way ANOVA, Tukey’s multiple comparison, *p <0.05, ***p <0.001, ns, not significant.

Aminoglycoside treatment has been shown to downregulate enzymes involved in sialylation in human podocytes (24, 25). Therefore, we analyzed the transcript levels of several genes involved in de- and re-sialylation, including glucosamine-2-epimerase/N-acetylmannosamine kinase (*Gne*), neuraminidases 1 and 3 (*Neu1* and *Neu3*), sialyltransferases ST3 beta-galactoside alpha-2,3-sialyltransferase 5 (*St3gal5*), ST6 beta-galactoside alpha-2,6-sialyltransferase 1 (*St6gal1*), ST6 N-acetylgalactosaminide alpha-2,6-sialyltransferase 2 (*St6galnac2*), and polysialyltransferase ST8 alpha-N-acetyl-neuraminide alpha-2,8-sialyltransferase 1 (*St8sia1*). Transcription of *Gne*, the essential enzyme in sialic acid biosynthesis, and transcription of *Neu1* were not affected by neomycin treatment or therapy with 6SL. However, we found increased transcription of neuraminidase 3 (*Neu3*) in the neomycin-treated group compared with the PBS control group (**Figure 5**). In detail, gene transcription of *Neu3* was increased from 0.86 ± 0.4 FC to 2.43 ± 1.7 FC in the Neo group compared to the control group (p = 0.041), while 6SL tended to revert this increase to 1.17 ± 0.6 FC in the Neo + 6SL group (p = 0.127 Neo + 6SL vs. Neo group). Interestingly, the gene transcripts of two sialyltransferases (*St3gal5* and *St6gal1*) were upregulated after Neo + 6SL treatment (**Figure 5**).

In summary, the data showed an increase after Neo treatment in the transcription of neuraminidase *Neu3*, an enzyme involved in desialylation, and a Neo+6SL-dependent increase in sialyltransferase *St3gal5* and *St6gal1* gene transcription.

### 3.5 6’-sialyllactose inhibited inflammatory and neurotoxic effects of human macrophages

**Figure 6:**
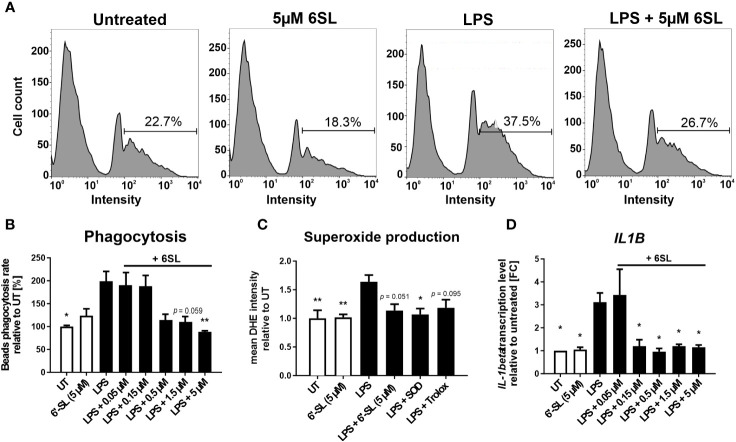
Anti-inflammatory and anti-oxidative effects of 6’-sialyllactose on cultured human THP1 macrophages. **(A)** Phagocytic uptake of microbeads by THP1 macrophages was analyzed using flow cytometry. A representative flow cytometric analysis of the four experimental groups is shown. The peak at the lowest intensity in each graph (intensity 1–10) represents the cells with no beads, and the peak at the next highest intensity (~40–80) represents the cells having taken up one bead. The percentage of cells with two or more beads was determined (percentage of cells showing an intensity of >90). LPS treatment was used to activate the phagocytic activity of THP1 macrophages. 6SL reduces the LPS-triggered phagocytic activity of THP1 macrophages. **(B)** Quantification of the phagocytic uptake of microbeads by THP1 macrophages was normalized to the untreated (UT) condition and revealed an increase after LPS treatment. Parallel treatment with 5 µM 6SL reduced LPS-induced increase in bead phagocytosis by human macrophages (THP1). Data are shown as mean ± SEM; n = 4–11; one-way ANOVA, Dunnett’s T3 *post-hoc* comparison; *p ≤0.05, **p ≤0.01. **(C)** Superoxide production was determined using dihydroethidium (DHE). Flow cytometry of human macrophages (THP1) showed increased superoxide production after lipopolysaccharide (LPS) stimulation. Treatment with 6SL tended to prevent LPS-triggered superoxide production to a similar degree as the antioxidant superoxide dismutase 1 (SOD) and the vitamin E analog Trolox. Data are shown as the mean ± SEM; n = 5; one-way ANOVA, Tukey *post-hoc* comparison, *p ≤0.05, **p ≤0.01. **(D)** Human macrophages (THP1) were stimulated with lipopolysaccharide (LPS) for 24 h. Transcription of the proinflammatory marker interleukin 1β (*IL1B*) was upregulated after LPS treatment, whereas simultaneous 6SL treatment attenuated the LPS-induced inflammatory effect. Data are shown as the mean ± SEM; n = 3–9; one-way ANOVA, Dunnett’s T3 *post-hoc* comparison, *p ≤0.05.

As our data indicated an inflammation-related mechanism of 6SL, we analyzed the effect of 6SL on human THP1 macrophages *in vitro*. Upon inflammatory stimulation with lipopolysaccharides (LPS), phagocytic activity increases in macrophages. Accordingly, we analyzed the effect of 6SL on the phagocytosis of beads by human THP1 macrophages challenged with LPS. The THP1 cells were differentiated into macrophage-like cells and stimulated with LPS. Using a bead phagocytosis assay, the percentage of cells that phagocytosed two or more beads was determined by flow cytometry (**Figure 6A**). While 6SL alone did not affect baseline phagocytosis, 6SL was able to reduce the LPS-increased phagocytosis rate of human macrophages in a dose-dependent manner (**Figure 6B**). In detail, LPS stimulation increased the phagocytic uptake of THP1 cells from 100% in untreated cells to 199.07% ± 71.33% (p = 0.020), whereas the addition of 5 µM 6SL alone (control) resulted in 122.79% ± 53.11% of phagocytosis rate. The LPS-induced increase in phagocytosis was reverted after treatment of macrophages with 0.05µM 6SL (189.41% ± 57.14%), with 0.15 µM 6SL (187.24% ± 49.27%), with 0.5µM 6SL (113.27% ± 43.64%), with 1.5µM 6SL (109.31% ± 33.76%; p = 0.059) or with 5µM 6SL (87.45% ± 8.68%; p = 0.008).

Another important defense mechanism of activated phagocytes is the release of reactive oxygen species (ROS), which is also triggered in macrophages cultured with LPS. In this study, 6SL was able to prevent the LPS-triggered oxidative burst of human macrophages, similar to the anti-oxidative enzyme superoxide dismutase 1 (SOD1) and the radical scavenger Trolox, a vitamin E analog (**Figure 6C**). In detail, the relative mean staining intensity of the superoxide marker dihydroethidium (DHE) was increased from 1.0 ± 0.3 in untreated THP1 cells to 1.64 ± 0.26 in cells stimulated with 3 µg/ml LPS (p = 0.008). The addition of 5 µM 6SL alone did not result in a change in the relative DHE staining intensity (1.02 ± 0.1). Treatment with 5 µM 6SL in LPS-stimulated cells reverted the relative DHE intensity to 1.14 ± 0.25 (vs. LPS; p = 0.051), similarly as the addition of the anti-oxidative enzyme SODs (1.07 ± 0.23; vs. LPS, p = 0.020) and the radical scavenger Trolox (1.18 ± 0.3; vs. LPS, p = 0.095).

Next, we analyzed the effect of 6SL on the transcription of inflammatory genes in human THP1 macrophages (**Figure 6D**). Transcription level of the pro-inflammatory cytokine interleukin 1β (*IL1B*) was increased after LPS stimulation to 3.12 ± 1.20 FC (p = 0.014) from 1.0 FC in untreated cells. Treatment with 6SL reduced the *IL1B* gene transcription from 3.12 ± 1.20 FC to 1.20 ± 0.55 FC (+ 0.15 µM 6SL; p = 0.047), to 0.96 ± 0.32 FC (+ 0.5 µM 6SL; p = 0.011), to 1.20 ± 0.15 FC (+ 1.5 µM 6SL; p = 0.024) and to 1.15 ± 0.18 FC (+ 5 µM 6SL; p = 0.021).

**Figure 7:**
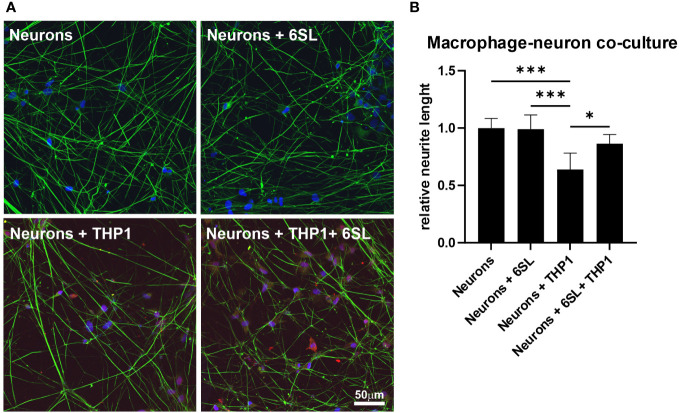
Neuroprotection of sensory neurons against macrophage-mediated neurite loss by 6’-sialyllactose. **(A)** Human sensory neurons (shown in green) were obtained from human induced pluripotent stems (iPSCs) and co-cultured with human macrophages (THP1, shown in red). Neurons or neurons co-cultured with THP1 macrophages were treated with 6’-Sialyllactose (6SL). The cellular nuclei were stained with DAPI (blue). The addition of THP1 macrophages to sensory neurons reduced overall neurite length. Scale bar: 50 µM. **(B)** Quantification of neurite length revealed no effect of 6SL treatment on the relative neurite length, while the addition of THP1 cells reduced the relative neurite length. Treatment with 6SL prevented the loss of neurite length in a co-culture of neurons and macrophages. Data are presented as mean ± SD, and data were collected from at least six images of repeated experiments; one-way ANOVA, Bonferroni *post-hoc* comparison, *p ≤0.05, ***p ≤0.001.

Microglia and macrophages remove debris or dying cells from their environment, thus helping to maintain homeostasis in the tissue. In this study, we mimicked the interplay of macrophages in the peripheral nervous system using human induced pluripotent stem cell (iPSC)-derived peripheral sensory neurons to understand the anti-inflammatory effect of 6SL in a more complex *in vitro* environment. While co-culturing sensory neurons with macrophages resulted in reduced neural branch length, 6SL treatment of sensory neurons without macrophages did not affect the neurite length. However, treatment with 6SL prevented the neurotoxic effects of human macrophages in co-culture with human iPSC-derived peripheral sensory neurons (**Figure 7**). In detail, the relative neural branch length (1.0 ± 0.08) was decreased to 0.64 ± 0.14 when co-cultured with THP1 macrophages (p ≤0.001) but was not affected when neurons were incubated with 6SL (0.99 ± 0.12), or less reduced when simultaneously treated with 6SL (0.86 ± 0.08; p = 2.77).

In summary, 6SL exhibited anti-inflammatory and neuroprotective effects in human macrophages.

## 4 Discussion

Aminoglycosides are potent antibiotics with nephrotoxic and ototoxic effects. Although their use has declined in industrialized countries with the emergence of cephalosporins, aminoglycosides have re-emerged for widespread use after the rise of multidrug-resistant bacteria (6). Neomycin belongs to the class of aminoglycosides that are highly nephrotoxic and ototoxic (26). In this study, we analyzed 6SL as a potential treatment for preventing aminoglycoside-related ototoxicity in a neomycin-induced hearing loss mouse model.

We observed that consecutive subcutaneous injections of neomycin resulted in ototoxicity, as determined by the elevated ABR thresholds (Neo group). The mouse cochlea is susceptible to aminoglycoside ototoxicity in the sensitive postnatal period up to an age of 20 days (27). Typically, neomycin-induced ototoxicity mainly targets the structures of the organ of Corti, particularly the hair cells, including their sensory neural innervations, starting with the outer hair cells, followed by the inner hair cells (28). Damage first occurs in the basal turn of the cochlea and then progresses to the middle and apical regions (29). Thus, hearing loss first occurs at higher tone frequencies (located in the basal region) and then extends to lower tone frequencies (29). ABR testing is an objective method for evaluating auditory pathways. In our study, we confirmed in our study, that neomycin application had a detrimental effect on the auditory pathways, resulting in elevated hearing thresholds 14 days after the end of treatment for all frequencies tested.

The ABR thresholds were less impaired when 6SL was applied simultaneously with neomycin as therapy (Neo + 6SL group). In particular, hearing thresholds were less elevated in the Neo + 6SL therapy group, but this effect varied between tone frequencies. For the highest frequency tested, we could not see a difference between the Neo and the Neo + 6SL therapy groups; threshold levels for lower frequencies were decreased after 6SL treatment and reached almost baseline levels (6 kHz). Because aminoglycoside ototoxicity is thought to be related to an oxidative burst, basal cells operating at higher frequencies appear to be highly susceptible to damage (30). Thus, it is possible that the damage to higher-frequency regions of the cochlea was too severe to be rescued by the protective effects of 6SL. Accordingly, we performed a more detailed analysis of the ABR wave I components, which have been described to be similar in small mammals and humans, and to reflect the VIII cranial nerve activity (31). The ABR thresholds were less elevated (**Figure 1C**) and ABR wave I latency reached control values at stronger activation (**Figure 1E**), suggesting that the activity in the peripheral auditory nerve of the Neo + 6SL group was less affected than that of the Neo group. It has been shown in age-related cochlear synaptopathy in mice that ABR wave I amplitudes correlate with the number of inner hair cell ribbons, while inner hair cell counts remain consistent across different ages (32). Thus, we cannot fully interpret our ABR wave I amplitude data without the quantification of hair cell numbers. Unfortunately, our histological analysis to quantify hair cell numbers in P28–P30 old mice failed because of insufficient preservation of the whole hair cell lining from the basal to the apical cochlea.

Macrophages are widely distributed in various sites of the cochlea, which are important for maintaining cochlear homeostasis (33, 34), similar to the homeostatic activity of microglia in the brain (35). Upon insult, cochlear macrophages become activated, changing their abundance, distribution, and morphology (35). Furthermore, macrophages also invade the cochlea from blood monocytes upon inner ear injury (36). Thus, the timely control of macrophage activation and/or invasion is necessary to limit inflammatory hair cell damage. There are different types of macrophages invading the inner ear, depending on the type of injury. In response to aminoglycoside ototoxicity, local CX3CR1 macrophages increase in number in the inner ear, while systemic LPS exposure increases the number of CCR2-expressing macrophages typically derived from blood monocytes (37). Studies have also shown that neomycin exposure leads to increased levels of inflammatory cytokines (7, 38).

Sialic acid-binding immunoglobulin-like lectin (Siglec) receptors sense sialylated glycostructures and are widely expressed in macrophages (39, 40). Most Siglec receptors mediate inhibitory signaling via their immunoreceptor tyrosine-based inhibition motif (ITIM) (41). Upon recognition of sialylation on neighboring cells by the Siglec receptors of mononuclear phagocytes, inhibitory Siglec-ITIM signaling leads to downregulation of the pro-inflammatory pathways in phagocytes (21, 42).

We demonstrated that 6SL dampened the cochlear macrophage immune response in neomycin-treated mice. Immunohistochemical evaluation showed increased macrophage numbers in the Rosenthal’s canal, the region where the cell bodies of the sensory neural innervations are located, in Neo-treated mice that were reverted to almost normal levels by 6SL (Neo + 6SL group). Data on inflammation-related *Aif1* and *Pik3cd* gene transcription in the whole cochlea supported the increased macrophage immune response observed in the spiral ganglion after Neo treatment, which was reversed by treatment with 6SL in the Neo + 6SL group. Several studies have elucidated the release of inflammatory mediators by activated macrophages associated with cochlear damage. Damaged tissues release damage-associated molecular patterns (DAMPs), which in turn act on pattern recognition receptors and result in a pro-inflammatory signature (34). Toll-like receptor 4 (TLR-4) is a pattern recognition receptor that is activated upon aseptic inflammation and leads to canonical activation of the NF-kB pathway, which subsequently promotes the release of pro-inflammatory cytokines such as Il-1β (34). In our study, the transcripts of the inflammatory marker *Aif1/Iba1* were elevated in the Neo group and returned to normal levels in the Neo + 6SL group. Likewise, transcripts of the pro-inflammatory cytokine Il-1β tended to increase in the Neo group, which was significantly returned to normal levels in the Neo + 6SL group, demonstrating the anti-inflammatory effect of 6SL.

An elevation in the ABR threshold in neomycin-treated mice, especially prominent at high frequencies, was described in a previous study (7). Sun et al. showed that in neomycin-treated mice, cochlear macrophages were recruited into the Organ of Corti, particularly in the middle and basal cochlear turns. Although treatment with the broad tetracycline antibiotic minocycline decreased the number of macrophages in the hair cell area and ABR thresholds in neomycin-treated mice, this treatment failed to rescue the outer and inner hair cell damage (7).

As a potential therapy, we tested whether the sialic acid carrier 6’-sialyllactose can ameliorate cochlear immune activation in neomycin-treated mice and protect against neomycin-induced hearing loss, as determined by measurement of ABR thresholds. In our study, we showed that 6SL therapy reverted the inner ear immune activation caused by neomycin insult, as reflected in the spiral ganglia by decreased macrophage number and/or activation, as well as lower pro-inflammatory gene transcription levels in the inner ear. We further showed that macrophage numbers reverted to normal levels in the therapy group (Neo + 6SL group), indicating a strong therapeutic effect similar to that of minocycline (7).

At the inner ear transcriptional level, we found an elevation in the apoptotic marker *Pik3cd* gene in the Neo-treated group, while the necroptotic markers showed no change between the different groups. In particular, apoptosis-related *Pik3cd* gene transcription was elevated in whole cochlea homogenates of neomycin-treated mice (Neo) compared to the PBS control group and was reversed after additional treatment with 6SL (Neo + 6SL). The *Pik3cd* gene contributes to the production of phosphatidylinositol 3-kinase (PI3K), which is expressed in macrophages and controls survival and apoptosis. While PI3K is undetectable in the resting state of cells, it substantially increases upon cellular stimulation (43). Furthermore, PI3K signaling was implicated in the promotion of hair cell survival after aminoglycoside exposure, and it has been shown that the inhibition of the PI3K signaling protein, PDK1, sensitized hair cells to gentamicin, but not to neomycin toxicity (44, 45). In line with our data, Ding et al. also reported that Fadd-mediated caspase-8 apoptosis does not play a role in aminoglycoside toxicity (46), which might explain why we have not observe an increase in other apoptotic markers such as Fadd and caspase-8.

To examine whether the complement system was involved in the Neo or the Neo + 6SL therapy group, we tested the transcription levels of different complement genes in the inner ear. Complement factor C3 is a critical node in all complement activation pathways. Complement factor C4b is only involved in the classical or lectin complement pathways (47). Interestingly, we found an elevation in the gene transcription of complement factor C4b in the Neo + 6SL therapy group, but there was no significant change in the C3 transcript among the three groups.

The mechanism underlying aminoglycoside-induced ototoxicity is not fully understood. The widely proposed mechanism involves the accumulation of reactive oxygen species (ROS) in the cochlea, which leads to apoptosis and cell death. Free radicals attack the cell membranes, proteins, and DNA, leading to lipid peroxidation, causing irreversible cell damage and cell death. Particularly, it has been shown that perivascular macrophage-like melanocytes in the lateral cochlear wall can produce radicals in response to injury, which in turn recruit blood monocytes (48). Thus, preventing oxidative stress might inhibit macrophage invasion from blood monocytes, and thereby could be helpful in preventing hearing loss, as elucidated earlier. Furthermore, the formation of iron-aminoglycoside complexes might be a possible factor leading to oxidative stress (49). Data from experimental and clinical studies have shown a positive effect of antioxidants, such as thymoquinone (50), aspirin (51), N-acetylcysteine, and vitamin A (52). In addition, flavonoids, such as naringenin, seem to reduce free radical formation after aminoglycoside treatment (53). However, to date, no agent has been approved for the clinical treatment of aminoglycoside-induced ototoxicity.

Sialic acid is known to scavenge free radicals locally (12, 54, 55). Sialic acid is a naturally occurring carbohydrate on our cellular glycocalyx, which is integrated in a terminal position in glycoproteins and glycolipids on the cell surface, acts as an immune checkpoint, and helps to maintain the immune system in a homeostatic state (10). Milk oligosaccharide 6’-sialyllactose is a natural sialic acid carrier that is already used in infants as a sialic acid source for early brain development, when an additional exogenous supply of sialic acid is needed to build up the glycocalyx of the brain (13).

To understand whether neomycin treatment or 6SL therapy interfered with the sialylation and desialylation process of the inner ear, we measured the transcriptional levels of different sialidases and sialyltransferases. While sialyltransferases add sialic acid residues to the carbohydrate chains of proteins and lipids in the endoplasmic reticulum, sialidases/neuraminidases remove sialic acids from glycoproteins and glycolipids (56). We observed increased neuraminidase 3 transcription in the Neo group, suggesting increased cleavage of sialic acids from gangliosides, which tended to revert in the Neo + 6SL treatment group. Interestingly, transcription of the ST6GAL1 sialyltransferase, which is an enzyme that typically catalyzes α2,6-sialylated milk and has activity towards lactose (57), was also increased in the Neo + 6SL group compared to the wild-type control and Neo group, suggesting an indirect effect on sialylation via 6SL uptake. In line with this finding, transcription of the ST3GAL5 sialyltransferase, which contributes to the formation of GM3 ganglioside, a precursor of several gangliosides (58) was also elevated in the Neo + 6SL group, while it remained unchanged in the Neo group compared to the wild-type control. It has been shown that GM3 is expressed in all cochlear regions, but only distinctly in the stria vascularis, spiral ganglion, and organ of Corti. GM3 synthase-deficient mice had a deformed organ of Corti and complete hearing loss, while maintaining normal stria vascularis function and normal structure of other cochlear regions (59). These data suggest that 6SL supplementation can indeed alter the cellular sialylation process and might contribute to increased formation of gangliosides, possibly allowing resialylation and strengthening of sialic acid checkpoints for immune cells and maintaining the structure of the organ of Corti.

In human podocytes, treatment with the aminonucleoside puromycin resulted in the loss of sialic acid from proteins, which was accompanied by a reduction in the expression of sialyltransferases. Interestingly, sialic acid supplementation rescued the sialylation of podocyte proteins and partially restored the expression of sialyltransferases (24).

*In vitro*, we were also able to demonstrate the dose-dependent anti-inflammatory and anti-phagocytic effects of 6SL on cultured human macrophages stimulated with lipopolysaccharide (LPS), a typical pro-inflammatory stimulus. Increasing concentrations of 6SL reduced the transcription of the pro-inflammatory marker Il-1β and phagocytic activity in LPS-treated human macrophages to levels similar to those in untreated cells. In both cases, 6SL alone had no effect on human macrophages. Moreover, we also showed the anti-oxidative effects of 6SL on cultured human macrophages, where it had similar effects on macrophages as the antioxidants superoxide dismutase 1 (SOD) and the vitamin E analog Trolox. Interestingly, it has been shown that SOD1 deficient mice show premature age-related hearing loss, and antioxidants such as vitamins C and E have hearing preservative effects (60). Thus, we showed a clear hallmark of a therapeutic approach that focuses on macrophages. Furthermore, we directly demonstrated the neuronal protective effect of 6SL on macrophage toxicity by showing neurite length preservation in an *in vitro* model of human macrophage-neuronal co-culture. Similar neuroprotective effects have been described with polymers of sialic acids with an average degree of polymerization of 20 (61), demonstrating the general anti-inflammatory and antioxidative properties of sialylated oligo/polysaccharides on macrophages.

In summary, neomycin leads to an increased ABR threshold in mice as a sign of functional hearing loss as well as increased inflammation in the inner ear. The results obtained in this study show that 6SL treatment can partially restore impaired hearing function in neomycin-treated mice and reverse inner ear inflammation. Our study also demonstrated the *in vitro* anti-inflammatory effects of 6SL on human macrophages as well as its anti-phagocytic and anti-oxidative properties, along with its protective ability in human neurons.

## Data availability statement

The original contributions presented in the study are included in the article/**Supplementary Material**. Further inquiries can be directed to the corresponding author.

## Ethics statement

Ethical approval was not required for the studies on humans in accordance with the local legislation and institutional requirements because only commercially available established cell lines were used. The animal study was approved by Dr. Laura Gey, Universitätsklinikum Bonn, Haus für Experimentelle Medizin (HET), Venusberg-Campus 1, 53127 Bonn, Germany. The study was conducted in accordance with the local legislation and institutional requirements.

## Author contributions

TA: Conceptualization, Investigation, Writing – original draft, Writing – review & editing, Methodology. TK: Methodology, Writing – original draft, Writing – review & editing, Investigation. TS: Investigation, Methodology, Writing – review & editing. AS: Investigation, Methodology, Writing – review & editing. HN: Writing – review & editing, Conceptualization, Funding acquisition, Project administration, Writing – original draft. CK: Conceptualization, Writing – original draft, Writing – review & editing, Data curation, Formal Analysis, Investigation, Project administration.

## References

[1] Wilson BS Tucci DL O’Donoghue GM Merson MH Frankish H. A Lancet Commission to address the global burden of hearing loss. Lancet (2019) 393(10186):2106–8. doi: 10.1016/S0140-6736(19)30484-2 30827785

[2] Bako P Gerlinger I Wolpert S Müller M Löwenheim H. The ototoxic effect of locally applied kanamycin and furosemide in Guinea pigs. J Neurosci Methods (2022) 372:109527. doi: 10.1016/J.JNEUMETH.2022.109527 35182603

[3] Yang C-H Schrepfer T Schacht J Coffin AB Steyger PS. Age-related hearing impairment and the triad of acquired hearing loss. Front Cell Neurosci (2015) 9:276. doi: 10.3389/fncel.2015.00276 26283913 PMC4515558

[4] Lee MY Park Y-H. Potential of gene and cell therapy for inner ear hair cells. BioMed Res Int (2018) 2018:1–11. doi: 10.1155/2018/8137614 PMC6020521 30009175

[5] Roth TN. Aging of the auditory system. *Handbook of Clinical Neurology* . 129 (2015) 129:357–73. doi: 10.1016/B978-0-444-62630-1.00020-2 25726279

[6] Krause KM Serio AW Kane TR Connolly LE. Aminoglycosides: an overview. Cold Spring Harbor Perspect Med (2016) 6(6):a027029. doi: 10.1101/CSHPERSPECT.A027029 PMC4888811 27252397

[7] Sun S Yu H Yu H Honglin M Ni W Zhang Y. Inhibition of the activation and recruitment of microglia-like cells protects against neomycin-induced ototoxicity. Mol Neurobiol (2014) 51(1):252–67. doi: 10.1007/s12035-014-8712-y 24781382

[8] Aran JM Darrouzet J Erre JP. Observation of click-evoked compound viii nerve responses before, during, and over seven months after kanamycin treatment in the Guinea pig. Acta Oto-Laryngologica (1975) 79:24–32. doi: 10.3109/00016487509124650 1096528

[9] Klaus C Liao H Allendorf DH Brown GC Neumann H. Sialylation acts as a checkpoint for innate immune responses in the central nervous system. Glia (2021) 69(7):1619–36. doi: 10.1002/GLIA.23945 33340149

[10] Liao H Klaus C Neumann H. Control of innate immunity by sialic acids in the nervous tissue. Int J Mol Sci (2020) 21(15):5494. doi: 10.3390/IJMS21155494 32752058 PMC7432451

[11] Shahraz A Lin Y Mbroh J Winkler J Liao H Lackmann M. Low molecular weight polysialic acid binds to properdin and reduces the activity of the alternative complement pathway. Sci Rep (2022) 12(1):5815. doi: 10.1038/S41598-022-09407-2 35388026 PMC8987038

[12] Iijima R Takahashi H Namme R Ikegami S Yamazaki M. Novel biological function of sialic acid (N-acetylneuraminic acid) as a hydrogen peroxide scavenger. FEBS Lett (2004) 561(1–3):163–6. doi: 10.1016/S0014-5793(04)00164-4 15013770

[13] Corona L Lussu A Bosco A Pintus R Marincola FC Fanos V. Human milk oligosaccharides: A comprehensive review towards metabolomics. Children (2021) 8(9):804. doi: 10.3390/CHILDREN8090804 34572236 PMC8465502

[14] Röhrig CH Choi SSH Baldwin N. The nutritional role of free sialic acid, a human milk monosaccharide, and its application as a functional food ingredient. Critic Rev Food Sci Nutrit (2017) 57(5):1017–38. doi: 10.1080/10408398.2015.1040113 26115419

[15] Nöhle U Schauer R. Metabolism of sialic acids from exogenously administered sialyllactose and mucin in mouse and rat. Hoppe-Seyler’s Z Fur Physiologische Chemie (1984) 365(12):1457–68. doi: 10.1515/BCHM2.1984.365.2.1457 6526381

[16] Phipps KR Baldwin NJ Stannard DR Šoltésová A Gilby B Mikš MH. Toxicological safety evaluation of the human-identical milk oligosaccharide 6′-sialyllactose sodium salt. J Appl Toxicol (2019) 39:10. doi: 10.1002/jat.3830 31389052

[17] Strenzke N Chakrabarti R Al-Moyed H Müller A Hoch G Pangrsic T. Hair cell synaptic dysfunction, auditory fatigue and thermal sensitivity in otoferlin Ile515Thr mutants. EMBO J (2016) 35(23):2519–35. doi: 10.15252/EMBJ.201694564 PMC5283603 27729456

[18] Rüttiger L Singer W Panford-Walsh R Matsumoto M Lee SC Zuccotti A. The reduced cochlear output and the failure to adapt the central auditory response causes tinnitus in noise exposed rats. PloS One (2013) 8(3):e57247. doi: 10.1371/JOURNAL.PONE.0057247 23516401 PMC3596376

[19] Suthakar K Liberman MC. A simple algorithm for objective threshold determination of auditory brainstem responses. Hearing Res (2019) 381:107782. doi: 10.1016/J.HEARES.2019.107782 PMC6726521 31437652

[20] Montgomery SC Cox BC. Whole mount dissection and immunofluorescence of the adult mouse cochlea. J Visualized Experiments: JoVE (2016) 107:53561. doi: 10.3791/53561 PMC4780967 26779585

[21] Klaus C Hansen JN Ginolhac A Gérard D Gnanapragassam VS Horstkorte R. Reduced sialylation triggers homeostatic synapse and neuronal loss in middle-aged mice. Neurobiol Aging (2020) 88:91–107. doi: 10.1016/J.NEUROBIOLAGING.2020.01.008 32087947

[22] Aminoff D. Methods for the quantitative estimation of N-acetylneuraminic acid and their application to hydrolysates of sialomucoids. Biochem J (1961) 81(2):384–92. doi: 10.1042/bj0810384 PMC1243351 13860975

[23] Chambers SM Qi Y Mica Y Lee G Zhang XJ Niu L. Combined small-molecule inhibition accelerates developmental timing and converts human pluripotent stem cells into nociceptors. Nat Biotechnol (2012) 30(7):715–20. doi: 10.1038/NBT.2249 PMC3516136 22750882

[24] Pawluczyk IZA Ghaderi Najafabadi M Patel S Desai P Vashi D Saleem MA. Sialic acid attenuates puromycin aminonucleoside-induced desialylation and oxidative stress in human podocytes. Exp Cell Res (2014) 320(2):258–68. doi: 10.1016/j.yexcr.2013.10.017 24200502

[25] Pawluczyk IZA Najafabadi MG Brown JR Bevington A Topham PS. Sialic acid supplementation ameliorates puromycin aminonucleoside nephrosis in rats. Lab Invest (2015) 95(9):1019–28. doi: 10.1038/LABINVEST.2015.78 26121320

[26] Macdonald RH Beck M. Neomycin: a review with particular reference to dermatological usage. Clin Exp Dermatol (1983) 8(3):249–58. doi: 10.1111/J.1365-2230.1983.TB01777.X 6224608

[27] Wu WJ Sha SH McLaren JD Kawamoto K Raphael Y Schacht J. Aminoglycoside ototoxicity in adult CBA, C57BL and BALB mice and the Sprague–Dawley rat. Hearing Res (2001) 158(1–2):165–78. doi: 10.1016/S0378-5955(01)00303-3 11506949

[28] Xie J Talaska AE Schacht J. New developments in aminoglycoside therapy and ototoxicity. Hearing Res (2011) 281(1–2):28–37. doi: 10.1016/J.HEARES.2011.05.008 PMC3169717 21640178

[29] Schacht J. Aminoglycoside ototoxicity: prevention in sight? Otolaryngology–Head Neck Surg (1998) 118(5):674–7. doi: 10.1177/019459989811800518 9591868

[30] Huth ME Ricci AJ Cheng AG. Mechanisms of aminoglycoside ototoxicity and targets of hair cell protection. Int J Otolaryngol (2011) 2011:1–19. doi: 10.1155/2011/937861 PMC3202092 22121370

[31] Allen AR Starr A. Auditory brain stem potentials in monkey (M. Mulatta) and man. Electroencephalography Clin Neurophysiol (1978) 45(1):53–63. doi: 10.1016/0013-4694(78)90341-3 78822

[32] Sergeyenko Y Lall K Charles Liberman M Kujawa SG. Age-related cochlear synaptopathy: an early-onset contributor to auditory functional decline. J Neurosci (2013) 33(34):13686–94. doi: 10.1523/JNEUROSCI.1783-13.2013 PMC3755715 23966690

[33] Hu BH Zhang C Frye MD. Immune cells and non-immune cells with immune function in mammalian cochleae. Hearing Res (2018) 362:14–24. doi: 10.1016/J.HEARES.2017.12.009 PMC5911222 29310977

[34] Zhang Y Li Y Fu X Wang P Wang Q Meng W. The detrimental and beneficial functions of macrophages after cochlear injury. Front Cell Dev Biol (2021) 9:631904. doi: 10.3389/FCELL.2021.631904 34458249 PMC8385413

[35] Hough K Verschuur CA Cunningham C Newman TA. Macrophages in the cochlea; an immunological link between risk factors and progressive hearing loss. Glia (2022) 70(2):219–38. doi: 10.1002/GLIA.24095 34536249

[36] Sato E Shick HE Ransohoff RM Hirose K. Expression of fractalkine receptor CX3CR1 on cochlear macrophages influences survival of hair cells following ototoxic injury. JARO: J Assoc Res Otolaryngol (2010) 11(2):223. doi: 10.1007/S10162-009-0198-3 19936834 PMC2862920

[37] Hirose K Li SZ Ohlemiller KK Ransohoff RM. Systemic lipopolysaccharide induces cochlear inflammation and exacerbates the synergistic ototoxicity of kanamycin and furosemide. JARO: J Assoc Res Otolaryngol (2014) 15(4):555. doi: 10.1007/S10162-014-0458-8 24845404 PMC4141430

[38] Limatola C Ransohoff RM. Modulating neurotoxicity through CX3CL1/CX3CR1 signaling. Front Cell Neurosci (2014) 8:229(AUG). doi: 10.3389/FNCEL.2014.00229 25152714 PMC4126442

[39] MacAuley MS Crocker PR Paulson JC. Siglec-mediated regulation of immune cell function in disease. Nat Rev Immunol (2014) 14(10):653–66. doi: 10.1038/NRI3737 PMC4191907 25234143

[40] Tateyama H Murase Y Higuchi H Inasaka Y Kaneoka H Iijima S. Siglec-F is induced by granulocyte-macrophage colony-stimulating factor and enhances interleukin-4-induced expression of arginase-1 in mouse macrophages. Immunology (2019) 158(4):340–52. doi: 10.1111/IMM.13121 PMC6856935 31520477

[41] Crocker PR Paulson JC Varki A. Siglecs and their roles in the immune system. Nat Rev Immunol (2007) 7(4):255–66. doi: 10.1038/NRI2056 17380156

[42] Lübbers J Rodríguez E van Kooyk Y. Modulation of immune tolerance via siglec-sialic acid interactions. Front Immunol (2018) 9:2807. doi: 10.3389/FIMMU.2018.02807 30581432 PMC6293876

[43] Rommel C Camps M Ji H. PI3K delta and PI3K gamma: partners in crime in inflammation in rheumatoid arthritis and beyond? Nat Rev Immunol (2007) 7(3):191–201. doi: 10.1038/NRI2036 17290298

[44] Jadali A Kwan KY. Activation of PI3K signaling prevents aminoglycoside-induced hair cell death in the murine cochlea. Biol Open (2016) 5(6):698–708. doi: 10.1242/BIO.016758 27142333 PMC4920183

[45] Wiedenhoft H Hayashi L Coffin AB. PI3K and inhibitor of apoptosis proteins modulate gentamicin- induced hair cell death in the zebrafish lateral line. Front Cell Neurosci (2017) 11:326. doi: 10.3389/FNCEL.2017.00326 29093665 PMC5651234

[46] Ding D Zhang J Jiang H Xuan W Qi W Salvi R. Some ototoxic drugs destroy cochlear support cells before damaging sensory hair cells. Neurotoxicity Res (2020) 37(3):743. doi: 10.1007/S12640-020-00170-8 PMC7065960 31997155

[47] Noris M Remuzzi G. Overview of complement activation and regulation. Semin Nephrol (2013) 33(6):479–92. doi: 10.1016/J.SEMNEPHROL.2013.08.001 PMC3820029 24161035

[48] Dai M Yang Y Omelchenko I Nuttall AL Kachelmeier A Xiu R. Bone marrow cell recruitment mediated by inducible nitric oxide synthase/stromal cell-derived factor-1alpha signaling repairs the acoustically damaged cochlear blood-labyrinth barrier. Am J Pathol (2010) 177(6):3089–99. doi: 10.2353/AJPATH.2010.100340 PMC2993278 21057001

[49] Sedó-Cabezón L Boadas-Vaello P Soler-Martín C Llorens J. Vestibular damage in chronic ototoxicity: a mini-review. Neurotoxicology (2014) 43:21–7. doi: 10.1016/J.NEURO.2013.11.009 24333467

[50] Sagit M Korkmaz F Gürgen SG Kaya M Akcadag A Ozcan I. The protective role of thymoquinone in the prevention of gentamicin ototoxicity. Am J Otolaryngol (2014) 35(5):603–9. doi: 10.1016/J.AMJOTO.2014.07.002 25087465

[51] Sha S-H Qiu J-H Schacht J. Aspirin to prevent gentamicin-induced hearing loss. New Engl J Med (2006) 354(17):1856–7. doi: 10.1056/NEJMC053428 16641409

[52] Aladag I Guven M Songu M. Prevention of gentamicin ototoxicity with N-acetylcysteine and vitamin A. J Laryngology Otology (2016) 130(5):440–6. doi: 10.1017/S0022215116000992 27095551

[53] Koçak S Aydogan E Şentürk E Akakın D Koroglu K Özer F. Evaluation of the possible protective role of naringenin on gentamicin-induced ototoxicity: A preliminary study. Int J Pediatr Otorhinolaryngology (2017) 100:247–53. doi: 10.1016/J.IJPORL.2017.07.008 28802382

[54] Eguchi H Ikeda Y Koyota S Honke K Suzuki K Gutteridge JMC. Oxidative damage due to copper ion and hydrogen peroxide induces GlcNAc-specific cleavage of an Asn-linked oligosaccharide. J Biochem (2002) 131(3):477–84. doi: 10.1093/OXFORDJOURNALS.JBCHEM.A003124 11872178

[55] Goswami K Nandakumar DN Koner BC Bobby Z Sen SK. Oxidative changes and desialylation of serum proteins in hyperthyroidism. Clinica Chimica Acta (2003) 337(1–2):163–8. doi: 10.1016/j.cccn.2003.08.009 14568194

[56] Rodriguez-Walker M Daniotti JL. Human sialidase neu3 is S-acylated and behaves like an integral membrane protein. Sci Rep (2017) 7(1):4167. doi: 10.1038/S41598-017-04488-W 28646141 PMC5482835

[57] Weiss GA Hennet T. The role of milk sialyllactose in intestinal bacterial colonization. Adv Nutr (Bethesda Md.) (2012) 3(3):483S–488S. doi: 10.3945/AN.111.001651 PMC3649486 22585928

[58] Gordon-Lipkin E Cohen JS Srivastava S Soares BP Levey E Fatemi A. ST3GAL5-related disorders: A deficiency in ganglioside metabolism and a genetic cause of intellectual disability and choreoathetosis. J Child Neurol (2018) 33(13):825–31. doi: 10.1177/0883073818791099 PMC6188822 30185102

[59] Yoshikawa M Go S Takasaki K Kakazu Y Ohashi M Nagafuku M. Mice lacking ganglioside GM3 synthase exhibit complete hearing loss due to selective degeneration of the organ of Corti. Proc Natl Acad Sci United States America (2009) 106(23):9483–8. doi: 10.1073/PNAS.0903279106 PMC2695060 19470479

[60] Kishimoto-Urata M Urata S Fujimoto C Yamasoba T. Role of oxidative stress and antioxidants in acquired inner ear disorders. Antioxidants (Basel Switzerland) (2022) 11(8):1469. doi: 10.3390/ANTIOX11081469 36009187 PMC9405327

[61] Shahraz A Kopatz J Mathy R Kappler J Winter D Kapoor S. Anti-inflammatory activity of low molecular weight polysialic acid on human macrophages. Sci Rep (2015) 5:16800. doi: 10.1038/srep16800 26582367 PMC4652165

