# Supplementary material for: 6’-sialyllactose ameliorates the ototoxic effects of the aminoglycoside antibiotic neomycin in susceptible mice
Source: Front Immunol. 2023 Dec 7;14:1264060. doi: 10.3389/fimmu.2023.1264060 (PMC10733791; doi:10.3389/fimmu.2023.1264060)
Supplement: Supplementary file 1 [file Table_1.docx]

Supplementary Material

Supplementary Table 1. Weight of analyzed mice

| **Group** | **Weight**  **(mean±SD)** | **One-way Anova** |
| --- | --- | --- |
| **For electrophysiology and immunohistochemistry** | | |
| **PBS (n=11)** | 13.5±3.1 g | P=0.522 |
| **Neo (n=5)** | 15.0±0.6 g |  |
| **Neo + 6SL (n=5)** | 13.9±1.1 g |  |
| **For transcriptomics** | | |
| **PBS (n=8)** | 10.8±5.3 g | P=0.899 |
| **Neo (n=8)** | 10.6±5.3 g |  |
| **Neo + 6SL (n=8)** | 9.7±4.5 g |  |
