# Supplementary material for: 6’-sialyllactose ameliorates the ototoxic effects of the aminoglycoside antibiotic neomycin in susceptible mice
Source: Front Immunol. 2023 Dec 7;14:1264060. doi: 10.3389/fimmu.2023.1264060 (PMC10733791; doi:10.3389/fimmu.2023.1264060)
Supplement: Supplementary file 2 [file Table_2.docx]

Supplementary Material

Supplementary Table 2. Pairwise comparisons of tone ABR thresholds

| **Frequency** | **Group** | **vs. Group** | **p-value, level** |
| --- | --- | --- | --- |
| **3 kHz** | PBS | Neo | 0.00031, *** |
|  | PBS | 6SL | 0.069, n.s. |
|  | Neo | 6SL | 0.96, n.s. |
| **6 kHz** | PBS | Neo | 2.4e-7, *** |
|  | PBS | 6SL | 0.429, n.s. |
|  | Neo | 6SL | 0.0033, ** |
| **12 kHz** | PBS | Neo | 2.24e-7, *** |
|  | PBS | 6SL | 0.00018, *** |
|  | Neo | 6SL | 0.00095, *** |
| **24 kHz** | PBS | Neo | 2.24e-7, *** |
|  | PBS | 6SL | 2.24e-7, *** |
|  | Neo | 6SL | 0.995, n.s. |
| F(Frequency)=17,9, p(Frequency)=8.1e-9 | | | |
| F(Treatment)=158.2, p(Treatment)=4.4e-27 | | | |
| F(Frequency*Treatment)=11.2, p(Frequency*Treatment)=8.3e-9 | | | |
| N=11,5,5 *4 | | | |
| df(total)=83 | | | |
