# Supplementary material for: 6’-sialyllactose ameliorates the ototoxic effects of the aminoglycoside antibiotic neomycin in susceptible mice
Source: Front Immunol. 2023 Dec 7;14:1264060. doi: 10.3389/fimmu.2023.1264060 (PMC10733791; doi:10.3389/fimmu.2023.1264060)
Supplement: Supplementary file 3 [file Table_3.docx]

Supplementary Material

Supplementary Table 3. Specific mouse oligonucleotides

| **Target** | **Orientation** | **Sequence (5’-3’)** |
| --- | --- | --- |
| Aif-1 | forward | GAAGCGAATGCTGGAGAAAC |
|  | reverse | AAGATGGCAGATCTCTTGCC |
| C3 | forward | TAGTGCTACTGCTGCTGTTGGC |
|  | reverse | GCTGGAATCTTGATGGAGACGCTT |
| C4b | forward | TGGAGGACAAGGACGGCTA |
|  | reverse | GGCCCTAACCCTGAGCTGA |
| Casp8 | forward | TGGAGAAGAGGACCATGCTG |
|  | reverse | AGTCACACAGTTCCGCCATT |
| Cd68 | forward | CAGGGAGGTTGTGACGGTAC |
|  | reverse | GAAACATGGCCCGAAGTATC |
| Cyba | forward | CCTCCACTTCCTGTTGTCGG |
|  | reverse | TCACTCGGCTTCCTTTCGGAC |
| Cybb | forward | GGGAACTGGGCTGTGAATGA |
|  | reverse | CAGTGCTGACCCAAGGAGTT |
| Dap12 | forward | ACAGCGGAAGGGACCCGGAAA |
|  | reverse | TCAGGCCGCTGATGGGCATA |
| Fcer1g | forward | CTGTCTACACGGGCCTGAAC |
|  | reverse | AAAGAATGCAGCCAAGCACG |
| Fadd | forward | GTGGCCTGGACCTGTTCAC |
|  | reverse | GGGCCAGTCTTTTCCAGTCT |
| Gapdh | forward | ACAACTTTGGCATTGTGGAA |
|  | reverse | GATGCAGGGATGATGTTCTG |
| Gne | forward | AAACTGGCCCCGATCATGTT |
|  | reverse | TCTACCATGGCCGCTTCATC |
| Inos | forward | AAGCCCCGCTACTACTCCAT |
|  | reverse | GCTTCAGGTTCCTGATCCAA |
| Il1β | forward | CTTCCTTGTGCAAGTGTCTG |
|  | reverse | CAGGTCATTCTCATCACTGTC |
| Mlkl | forward | CAAACAGTGAAGCCCCCTGA |
|  | reverse | AGCTGCTGATGTTTCTGTGGA |
| Neu1 | forward | TGGACCTGGCTCAGGCATTC |
|  | reverse | AAGCTCGTAGGGCTGGCAC |
| Neu3 | forward | CCTCCGGAGCCGAAGCC |
|  | reverse | GGGTGGGAGGAAGGTACAGC |
| Pik3cd | forward | GTCCACTCCTCCTCCATCCT |
|  | reverse | CAGCATTCACTTTTCGGCCC |
| Ripk1 | forward | TGTACCCTTACCTCCGAGCA |
|  | reverse | GGCTGCGGTTTTGTCTGTTT |
| Tnfα | forward | GGTGCCTATGTCTCAGCCTC |
|  | reverse | TGAGGGTCTGGGCCATAGAA |
| St3gal5 | forward | GGTGTTGAGGTGGGAGGAGAG |
|  | reverse | GATGGACTAGCAGAAAGGGGTTATGAA |
| St6gal1 | forward | CTAGACGGGGACGTATCGGAG |
|  | reverse | GGATGATCAAAAACCATCTCAGCA |
| St6galnac2 | forward | TCATGCTGTACTCCTCGGCG |
|  | reverse | TGGCAAAGTCGGCTCTTTCTG |
| St8sia1 | forward | GTGGCTGTGGCCGTCAAATA |
|  | reverse | GCAGGTTTTCAAATCTCTGGCG |
