# Supplementary material for: 6’-sialyllactose ameliorates the ototoxic effects of the aminoglycoside antibiotic neomycin in susceptible mice
Source: Front Immunol. 2023 Dec 7;14:1264060. doi: 10.3389/fimmu.2023.1264060 (PMC10733791; doi:10.3389/fimmu.2023.1264060)
Supplement: Supplementary file 4 [file Table_4.docx]

Supplementary Material

Supplementary Table 4: Specific human oligonucleotides

| **Target** | **Orientation** | **Sequence** |
| --- | --- | --- |
| GAPDH | forward | CTGCACCACCAACTGCTTAG |
|  | reverse | TTCAGCTCAGGGATGACCTT |
| IL1B | forward | CTTCCTTGTGCAAGTGTCTG |
|  | reverse | CAGGTCATTCTCATCACTGTC |
